# Comprehensive biomarker analysis of metabolomics in different syndromes in traditional Chinese medical for prediabetes mellitus

**DOI:** 10.1186/s13020-024-00983-1

**Published:** 2024-08-25

**Authors:** Qin Lan, Xue Li, Jianhe Fang, Xinyu Yu, Zhanxuan E. Wu, Caiyun Yang, Hui Jian, Fei Li

**Affiliations:** 1 Jiangxi University of Traditional Chinese Medicine, Nanchang, 330004 China; 2 Department of Gastroenterology and Hepatology, Laboratory of Metabolomics and Drug-Induced Liver Injury, Frontiers Science Center for Disease-Related Molecular Network, and State Key Laboratory of Respiratory Health and Multimorbidity, West China Hospital, Sichuan University, Chengdu, 610041 Sichuan China; 3 Medical Ancient Literature Teaching and Research Office, Jiangxi University of Traditional Chinese Medicine, Nanchang, 330004 China; 4 Discipline of Chinese and Western Integrative Medicine, Jiangxi University of Chinese Medicine, Nanchang, 330004 China; 5 Outpatient Department, Hongdu Traditional Chinese Medicine Hospital Affiliated to Jiangxi University of Traditional Chinese Medicine, Nanchang, 330006 China; 6 Endocrinology Department II, Hongdu Traditional Chinese Medicine Hospital Affiliated to Jiangxi University of Traditional Chinese Medicine, Nanchang, 330006 China

**Keywords:** Prediabetes mellitus, Spleen-dampness, Untargeted metabolomics, Insulin resistance, Obesity, Lipid metabolism

## Abstract

**Background:**

Prediabetes mellitus (PreDM) is a high-risk state for developing type 2 diabetes mellitus (T2DM) and often goes undiagnosed, which is closely associated with obesity and characterized by insulin resistance that urgently needs to be treated.

**Purpose:**

To obtain a better understanding of the biological processes associated with both "spleen-dampness" syndrome individuals and those with dysglycaemic control at its earliest stages, we performed a detailed metabolomic analysis of individuals with various early impairments in glycaemic control, the results can facilitate clinicians’ decision making and benefit individuals at risk.

**Methods:**

According to the diagnostic criteria of TCM patterns and PreDM, patients were divided into 4 groups with 20 cases, patients with syndrome of spleen deficiency with dampness encumbrance and PreDM (PDMPXSK group), patients with syndrome of dampness-heat in the spleen and PreDM (PDMSRYP group), patients with syndrome of spleen deficiency with dampness encumbrance and normal blood glucose (NDMPXSK group), and patients with syndrome of dampness-heat in the spleen and normal blood glucose (NDMSRYP group). Plasma samples from patients were collected for clinical index assessment and untargeted metabolomics using liquid chromatography-mass spectrometry.

**Results:**

Among patients with the syndrome of spleen deficiency with dampness encumbrance (PXSK), those with PreDM (PDMPXSK group) had elevated levels of 2-hour post-load blood glucose (2-h PG), glycosylated hemoglobin (HbA1c), high-density lipoprotein cholesterol (HDL-C), and systolic blood pressure (SBP) than those in the normal blood glucose group (NDMPXSK group, *P* < 0.01). Among patients with the syndrome of dampness-heat in the spleen (SRYP), the levels of body mass index (BMI), fasting blood glucose (FBG), 2-h PG, HbA1c, and fasting insulin (FINS) were higher in the PreDM group (PDMSRYP group) than those in the normal blood glucose group (NDMSRYP group, *P* < 0.05). In both TCM syndromes, the plasma metabolomic profiles of PreDM patients were mainly discriminatory from the normal blood glucose controls of the same syndrome in the levels of lipid species, with the PXSK syndrome showing a more pronounced and broader spectrum of alterations than the SRYP syndrome. Changes associated with PreDM common to both syndromes included elevations in the levels of 27 metabolites which were mainly lipid species encompassing glycerophospholipids (GPs), diglycerides (DGs) and triglycerides (TGs), cholesterol and derivatives, and decreases in 5 metabolites consisting 1 DG, 1 TG, 2 N,N-dimethyl phosphatidylethanolamine (PE-NMe2) and iminoacetic acid. Correlation analysis identified significant positive correlations of 3α,7α,12α,25-Tetrahydroxy-5β-cholestane-24-one with more than one glycaemia-related indicators, whereas DG (20:4/20:5) and PC (20:3/14:0) were positively and PC (18:1/14:0) was inversely correlated with more than one lipid profile-related indicators. Based on the value of correlation coefficient, the top three correlative pairs were TG with PC (18:1/14:0) (r = − 0.528), TG with TG (14:0/22:4/22:5) (r = 0.521) and FINS with PE-NMe (15:0/22:4) (r = 0.52).

**Conclusion:**

Our results revealed PreDM patients with different TCM syndromes were characterized by different clinical profiles. Common metabolite markers associated with PreDM shared by the two TCM syndromes were mainly lipid species encompassing GP, GL, cholesterol and derivatives. Our findings were in line with the current view that altered lipid metabolism is a conserved and early event of dysglycaemia. Our study also implied the possible involvement of perturbed bile acid homeostasis and dysregulated PE methylation during development of dysglycaemia.

**Supplementary Information:**

The online version contains supplementary material available at 10.1186/s13020-024-00983-1.

## Introduction

Prediabetes mellitus (PreDM) is an early stage of dysglycaemia control which can be categorized into impaired fasting glucose (IFG), impaired glucose tolerance (IGT), IFG combined with IGT (IFG + IGT), which is also a transitional stage between normal blood glucose and diabetes [1]. According to the IDF Diabetes Atlas 2021, about 170 million adults in China had IGT and 27 million adults had IFG in 2021 [2]. The prevalence of PreDM has been steadily rising. It has been estimated that the number of patients worldwide will reach 470 million in 2030, and the prevalence rate of PreDM in China might rise to as high as 35.2% [3]. PreDM represents a high-risk, yet often undiagnosed state for developing type 2 diabetes mellitus (T2DM) and its complications, as well as increasing the risk of cardiovascular and microvascular diseases, tumors, and Alzheimer's disease [4]. Prevention of the disease progression towards T2DM is possible with early detection and management, such as lifestyle interventions including diet, exercise, and weight loss. As such, PreDM represents a critical stage for the prevention and reversal of further worsening of dysglycaemia [5].

In Traditional Chinese Medicine (TCM), "spleen" does not refer to the anatomical spleen organ, but rather a system that controls the digestion, absorption, transformation, and metabolism of dietary substances, and its function is closely related to nutrient and energy metabolism of the body [6, 7]. The PreDM pathogenicity syndrome element involves both vacuity pattern and repletion pattern. While the syndrome element of repletion pattern is dominated by phlegm and dampness, the syndrome element of vacuity pattern is dominated by qi and yin deficiency. Among the complex combinations of the TCM syndrome element of PreDM, the "spleen-dampness" is of the highest support and confidence level [8]. Spleen-dampness is characterized by a heavy sensation in the head and body, lack of strength, lassitude and fatigue, gastric stuffiness, abdominal distension, nausea, and loose stool. Whilst PreDM is usually asymptomatic, classic signs suggestive of progression towards T2DM onset include fatigue, increased thirst and hunger, frequent urination and unintentional weight loss, etc. Fatigue and tiredness appears to be the only common clinical symptom between spleen-dampness and hyperglycaemia. Although loss of appetite is not a typical sign of T2DM, the sense of indigestion and nausea presented in spleen-dampness is somewhat similar to symptoms of a condition called diabetic gastroparesis in which the gastric emptying is delayed due to neuropathy in the setting of chronic hyperglycemia. "Spleen-dampness" is a stage in which the syndrome of spleen deficiency with dampness encumbrance is the main manifestations [9]. The pathologic product of dampness is well correlated with blood glucose level, and the evidence score of dampness is more informative than the blood glucose level in discriminating dysglycaemia patients from normoglycemic individuals [10]. Guidelines and expert consensus on the treatment of PreDM emphasize that the "spleen-damp" syndrome is critical for the TCM-based identification of PreDM. There are two patterns of "spleen-dampness" most frequently observed in PreDM, namely the syndrome of spleen deficiency with dampness encumbrance (PXSK) and the syndrome of dampness-heat in the spleen (SRYP) [11, 12]. Since patients of PXSK or SRYP display different types of pulses (as defined by TCM), clinical symptoms, and manifestations, yet it remains to be determined the shared and unique metabolic changes associated with PreDM in these patterns.

With the rapid advancements in analytical techniques, metabolomics is emerging as an attractive tool for biomarker discovery in diabetes and its complications, since metabolites can provide information about the molecular pathways involved in the development and progression of disease [13]. To gain deeper insights into the biological processes associated with individuals presenting "spleen-dampness" syndrome and those at the earliest stages of dysglycaemia, we performed a comprehensive metabolomic analysis of individuals with various early impairments in glucose control, the results can facilitate clinicians’ decision making and benefit individuals at risk.

## Materials and methods

### Research design and methods

From April 2023 to December 2023, patients with PreDM who attended the physical examination center and internal medicine clinic of Hongdu Hospital of Traditional Chinese Medicine in Nanchang City were selected for the study. According to the diagnostic criteria of TCM patterns and PreDM, these patients were divided into 4 groups of 20 cases: the patients with the syndrome of spleen deficiency with dampness encumbrance and PreDM (PDMPXSK group), the patients with the syndrome of dampness-heat in the spleen and PreDM (PDMSRYP group), the patients with the syndrome of spleen deficiency with dampness encumbrance and normal blood glucose (NDMPXSK group), and the patients with the syndrome of dampness-heat in the spleen and normal blood glucose (NDMSRYP group). Ethical approval for the study was obtained from the Medical Ethics Committee of the Hongdu Traditional Chinese Medicine Hospital, Nanchang, China (No. KYKS-2023194), and all participants provided signed informed consent forms.

The diagnostic criteria for PreDM in this study are based on the *Guideline for the Prevention and Treatment of Type 2 diabetes mellitus in China (2020 edition)* [1]. Patients with PreDM were identified by meeting the following criteria, (1) IFG: 6.1 ≤ fasting blood glucose (FBG) < 7.0 mmol/L, and 2-hour post-load blood glucose (2-h PG) < 7.8 mmol/L, (2) IGT: 7.8 ≤ 2-h PG < 11.1 mmol/L, with FBG < 7.0 mmol/L. (3) IFG + IGT: 6.1 ≤ FBG < 7.0 mmol/L and 7.8 ≤ 2-h PG < 11.1 mmol/L.

The diagnostic criteria for TCM patterns in PreDM are referenced from the *Guidelines for Evidence-based Clinical Practice of Traditional Chinese Medicine in Prediabetes* [12] and the *Clinic Terminology of Traditional Chinese Medical Diagnosis and Treatment—Part 2: Syndromes/Patterns* [14]. Patients with one of the following symptoms were recognized as dampness-heat in the spleen (SRYP): (1) Patients with two major clinical symptoms, with one minor clinical symptom, and with the tongue and pulse. (2) Patients with one major symptom, with three minor symptoms, and with the tongue and pulse. The major clinical symptoms include: fullness and distention of the abdomen, heavy and drowsiness of the body. The minor clinical symptoms include: dry mouth and thirst, or sweetness and greasiness in the mouth, eating less and lack of appetite, scanty and yellow urine, and loose stool. Tongue: red tongue, thick and greasy moss or slightly yellowish and not moist. Pulse: smooth pulse. At the same time, patients with the following symptoms were recognized as spleen deficiency with dampness encumbrance (PXSK): (1) Patients with two major clinical symptoms, with one minor clinical symptom, and with the tongue and pulse. (2) Patients with one major symptom, with three minor symptoms, and with the tongue and pulse. The major clinical symptoms include: obesity and abdominal hypertrophy, torpid intake and feel lassitude. The minor clinical symptoms include: sloppy stool, or tasteless and sticky in the mouth, heaviness in the limbs, tightness in the chest, and loose stool. Tongue: thin and white fur with teeth-printed. Pulse: moistened and slow pulse.

In short, patients were considered eligible if they met the following criteria: (1) Age of 18—65 years. (2) Fulfill the diagnostic criteria of the TCM symptoms for PreDM. (3) Patients gave their written consent to participate in the study, as well as, they were able to accept the questionnaire, and cooperated in the completion of relevant laboratory tests. Exclusion criteria included: (1) Patients with infectious diseases and severe trauma. (2) Pregnant women or lactating women. (3) Patients have severe heart, liver, renal insufficiency diseases, thyroid disorders, severe anemia, or malignant tumors. (4) Patients have taken drugs that interfere with blood sugar in the past 3 months [15].

### Clinical data

Clinical data collection mainly includes basic information, physical examination, information of inspection, listening and smelling examination, inquiry, palpation in TCM, and blood biochemical indices. Basic information including name, gender, age, occupation, place of residence, Personal history, family history of diabetes, history of hypertension, and other general conditions. Physical examination including height (Ht, cm), weight (Wt, kg), waist circumference (W, cm), hip circumference (H, cm), body mass index (BMI, %), and waist-to-hip ratio (WHR, %). TCM information including the dampness syndrome scale of Chinese Medicine [16], tongue manifestation, and pulse condition, their information was collected by 2 strictly trained and qualified Chinese medicine professionals according to the norms.

The blood lipid-related indexes include: blood routine examination, systolic blood pressure (SBP, mmHg), diastolic blood pressure (DBP, mmHg), FBG (mmol/L), 2-h PG (mmol/L), glycosylated hemoglobin (HbA1c, %), fasting insulin (FINS, μU/mL), total cholesterol (TC, mmol/L), triglyceride (TG, mmol/L), high-density lipoprotein cholesterol (HDL-C, mmol/L), low-density lipoprotein cholesterol (LDL-C, mmol/L) and uric acid (UA, mmol/L) using test kit and biochemical analyzer (BS-2000 M, Mindray company). Additionally, the values of homeostatic model assessment for insulin resistance (HOMA-IR), homeostatic model assessment for insulin sensibility (HOMA-IS), and homeostatic model assessment of β-cell function (HOMA-β) were calculated. The HOMA-IR was calculated according to the following formula: FINS (μU/mL) × FBG (mmol/L) / 22.5, HOMA-IS = 1 / HOMA-IR. The HOMA-β was calculated using the formula: 20 × FINS (μU/mL) / (FBG (mmol/L) – 3.5) [17].

Patients underwent fasting venous blood collection from 8:00–9:00 a.m. on the same day, they were instructed to eat a light diet for the previous 1 day and not to eat after 8:00 p.m.

### Sample preparation for LC–MS

Fifty microliter plasma sample was mixed with 300 μL of pre-chilled extraction solution (Acetonitrile: Methanol = 1:4, v/v, LC/MS, Merck) containing internal standards, followed by centrifugation at 12,000 rpm for 10 min at 4 °C. Two hundred microliter supernatant was transferred to a new tube and allowed to stand for 30 min at − 20 °C for protein precipitation. Subsequently, samples were centrifuged at 12,000 rpm for 3 min at 4 °C, 180 μL supernatant was transferred and filtered through a microporous membrane (0.22 μm pore size, Merck) into a vial for LC–MS analysis.

### LC–MS conditions

The liquid chromatography system was equipped with a Vanquish autosampler, a Vanquish detector, and a Vanquish pump (Thermo Fisher, USA), along with a Waters ACQUITY Premier HSS T3 Column (1.8 µm, 2.1 mm * 100 mm, Waters, USA). The mobile phase was 0.1% formic acid in water as solvent A and 0.1% formic acid (LC/MS, Aladdin) in acetonitrile as solvent B. The flow rate was 0.4 mL/min with the following gradient: 5% to 20% in 2 min, then increased to 60% in the following 3 min, increased to 99% in 1 min, and held at 99% for 1.5 min, then the gradient returned to 5% mobile phase B within 0.1 min and was held at the level for 2.4 min. The analytical conditions were as follows: the column temperature at 40 °C and the injection volume 4 μL. Another aliquot was analyzed under negative ion conditions using the same elution gradient as in the positive mode. Data acquisition was performed using the information-dependent acquisition (IDA) mode with Analyst TF 1.7.1 Software (Sciex, Concord, ON, Canada).

### Data processing and multivariate data analysis

The raw data files obtained from LC–MS were converted into mzXML format using ProteoWizard software. Peak extraction, peak alignment, and retention time correction were respectively performed utilizing the XCMS program. The peak areas were corrected with the "SVR" method, and peaks with a detection rate lower than 50% in each group of samples were discarded. Our metabolomics data acquisition and pre-processing were conducted by Wuhan Metaviral Biotechnology Co., Ltd. After that, metabolic identification information was obtained by searching the laboratory's self-built database (Wuhan Metaviral Biotechnology Co., Ltd.), the comprehensive public database (METLIN metabolite database (Metlin, http://metlin.scripps.edu/index.php), The Human Metabolome Database (HMDB database, https://hmdb.ca/)), and MetDNA (Metabolite identification and Dysregulated Network Analysis) [18]. Afterward, multi-factorial analyses were performed using the SIMCA 14.1 software package. Unsupervised principal component analysis (PCA) was performed using the statistical function prcomp within R (www.r-project.org). The data was unit variance scaled before unsupervised PCA [19].

The variable importance in projection (VIP) of the supervised orthogonal partial least-squares discriminant analysis (OPLS-DA) model was further used to search for potential plasma biomarkers. For two-group analysis, differential metabolites were determined by VIP (VIP > 1) and *P*-value (*P*-value < 0.05, Student’s t-test). For multi-group analysis, differential metabolites were determined by VIP (VIP > 1) and *P*-value (*P*-value < 0.05, ANOVA). The data was log transform (log_2_) and mean centering before OPLS-DA. Finally, to avoid overfitting, a permutation test was performed with 200 permutations to validate the stability of the results.

### Differential metabolite classification and Venn diagram analysis

Chemical classification of the identified metabolites was assigned using the HMDB database. Venn diagram analysis was employed to obtain common or unique metabolites associated with each condition (https://bioinformatics.psb.ugent.be/webtools/Venn/).

### KEGG annotation and enrichment analysis

Identified metabolites were annotated using the Kyoto Encyclopedia of Genes and Genomes (KEGG) Compound database (http://www.kegg.jp/kegg/compound/). The annotated metabolites were then mapped to the KEGG Pathway database (http://www.kegg.jp/kegg/pathway.html). Significantly enriched pathways were identified using a hypergeometric test, with a *P*-value for a given list of metabolites.

### Statistical analysis

Data was expressed as mean ± standard deviation (Mean ± SD). One-way analysis of variance (ANOVA) was performed using IBM SPSS Statistics 23.0 software (IBM, Armonk, NY, US). To determine the significance, statistical tests were used by the distribution of variables and the nature of the data (Student t-test, Mann–Whitney U test, Kruskal–Wallis test, Friedman test, F test). All of the comparison results were statistically significant (*P* < 0.05). GraphPad Prism 9.0 software (GraphPad Software, San Diego, California, US) was used for plotting.

## Results

### Baseline anthropometric and demographic characteristics of patients

Eighty subjects completed the study, 40 in the PXSK group (20 in normal blood glucose and 20 in PreDM) and 40 in the SRYP group (20 in normal blood glucose and 20 in PreDM). The results of the TCM patterns scoring criteria are provided in Table S1. The demographic and anthropometrical characteristics of the PXSK patients are presented in Table 1. No differences were observed in the age, BMI, or anthropometrical values between the subjects in the NDMPXSK and PDMPXSK groups. In contrast, the level of BMI was significantly higher in PreDM than the normal blood glucose group among the SRYP patients (*P* < 0.001) (Table 2).

**Table 1:** Baseline anthropometric and demographic characteristics of the PXSK population

	NDMPXSK n = 20 (Male: 9 Female: 11)	PDMPXSK n = 20 (Male: 9 Female: 11)	t / z	*p*	
Age (years)	46.78 ± 11.38	51.05 ± 9.75	1.274	0.210	n.s
Ht. (cm)	165.60 ± 8.11	163.50 ± 9.53	0.750	0.458	n.s
Wt. (kg)	65.25 ± 11.45	61.33 ± 13.96	0.971	0.338	n.s
BMI (%)	25.76 ± 5.41	22.81 ± 3.73	1.327	0.192	n.s
W (cm)	88.70 ± 14.13	82.62 ± 10.63	1.538	0.132	n.s
H (cm)	99.25 ± 12.62	92.53 ± 8.42	1.981	0.055	n.s
WHR (%)	0.87 ± 0.50	0.89 ± 0.06	0.178	0.860	n.s

*Ht* height, *Wt* weight, *BMI* body mass index, *W* waist circumference, *H* hip circumference, *WHR* waist-to-hip ratio, *n.s.* not significant. The Mann–Whitney U test was used to test for significant differences between the groups, data are n (%) or mean ± SD

**Table 2:** Baseline anthropometric and demographic characteristics of the SRYP population

	NDMSRYP n = 20 (Male: 9 Female: 11)	PDMSRYP n = 20 (Male: 12 Female: 8)	t / z	*p*	
Age (years)	40.65 ± 9.10	43.50 ± 7.88	1.059	0.296	n.s
Ht. (cm)	163.33 ± 6.53	165.35 ± 7.17	0.932	0.357	n.s
Wt. (kg)	64.67 ± 13.12	69.63 ± 9.84	1.353	0.184	n.s
BMI (%)	23.98 ± 3.43	32.71 ± 2.46	9.249	0.001	***
W (cm)	81.00 ± 10.14	87.95 ± 14.80	1.732	0.091	n.s
H (cm)	94.15 ± 6.46	94.99 ± 17.64	0.199	0.843	n.s
WHR (%)	0.85 ± 0.08	0.92 ± 0.78	0.399	0.692	n.s

*Ht* height, *Wt* weight, *BMI* body mass index, *W* waist circumference, *H* hip circumference, *WHR* waist-to-hip ratio, *n.s.* not significant, ***: *p* < 0.001. The Mann–Whitney U test was used to test for significant differences between the groups, data are n (%) or mean ± SD

### Comparison of glycolipid-related indicators in normal blood glucose group and PreDM group among the PXSK population

Among the PXSK patients, the PreDM group was characterized by significantly elevated SBP (*P* < 0.01), 2-h PG (*P* < 0.001), HbA1c (*P* < 0.001) and HDL-C (*P* < 0.001), and decreased UA level (*P* < 0.05). The corresponding results are shown in Table 3.

**Table 3:** Comparison of the glycolipid-related indicators between the normal blood glucose and PreDM group among the PXSK patients

	NDMPXSK n = 20 (Male: 9 Female: 11)	PDMPXSK n = 20 (Male: 9 Female: 11)	t / z	*p*	
SBP (mmHg)	112.10 ± 7.13	122.30 ± 11.50	3.371	0.002	**
DBP (mmHg)	73.85 ± 6.08	77.85 ± 10.07	1.521	0.137	n.s
FBG (mmol/L)	5.40 ± 0.39	5.82 ± 0.66	1.458	0.153	n.s
2-h PG (mmol/L)	5.57 ± 0.92	8.88 ± 1.26	9.489	0.001	***
HbA1c (%)	4.76 ± 0.37	5.46 ± 0.49	5.100	0.001	***
FINS (μU/mL)	8.04 ± 5.58	6.60 ± 3.92	0.944	0.351	n.s
UA (mmol/L)	363.90 ± 88.36	295.48 ± 104.47	2.236	0.031	*
TG (mmol/L)	1.33 ± 0.59	1.92 ± 1.48	1.660	0.106	n.s
TC (mmol/L)	4.63 ± 0.84	4.71 ± 1.05	0.266	0.792	n.s
HDL-C (mmol/L)	1.33 ± 0.28	1.65 ± 0.37	2.498	0.017	*
LDL-C (mmol/L)	2.78 ± 0.58	2.96 ± 0.84	0.789	0.435	n.s
HOMA-IR	1.91 ± 1.27	1.72 ± 1.09	0.508	0.615	n.s
HOMA-IS	0.68 ± 0.30	0.82 ± 0.49	1.090	0.283	n.s
HOMA-β (%)	93.40 ± 78.84	61.72 ± 42.87	1.579	0.123	n.s

*SBP* systolic blood pressure, *DBP* diastolic blood pressure, *FBG* fasting blood glucose, *2-h PG* 2-hour post-load blood glucose, *HbA1c* glycosylated hemoglobin, *FINS* fasting insulin, *UA* uric acid, *TG* triglyceride, *TC* total cholesterol, *HDL-C* high-density lipoprotein cholesterol, *LDL-C* low-density lipoprotein cholesterol, *HOMA-IR* homeostatic model assessment for insulin resistance, *HOMA-IS* homeostatic model assessment for insulin sensibility, *HOMA-β* homeostatic model assessment of β-cell function. *n.s.* not significant, *p < 0.05, **p < 0.01, ***p < 0.001. The Mann–Whitney U test was used to test for significant differences between the groups, data are n (%) or mean ± SD

### Comparison of Glycolipid-related indicators in normal blood glucose and PreDM groups among the SRYP population

Among the SRYP patients, the levels of FBG (*P* < 0.001), 2-h PG (*P* < 0.001) and HbA1c (*P* < 0.01), and FINS (*P* < 0.05) in PreDM group were higher than those in the normal blood glucose group (NDMSRYP group), and the level of HOMA-IS (*P* < 0.05) in PDMPXSK group was lower than that in NDMSRYP group (Table 4).

**Table 4:** Comparison of the glycolipid-related indicators between the normal blood glucose group and PreDM group among the SRYP patients

	NDMSRYP n = 20 (Male: 9 Female: 11)	PDMSRYP n = 20 (Male: 12 Female: 8)	t / z	*p*	
SBP (mmHg)	114.00 ± 13.24	120.20 ± 10.89	1.617	0.114	n.s
DBP (mmHg)	75.90 ± 10.18	78.40 ± 7.76	0.873	0.388	n.s
FBG (mmol/L)	5.35 ± 0.33	6.04 ± 0.55	4.811	0.001	***
2-h PG (mmol/L)	5.69 ± 1.13	9.12 ± 1.21	9.265	0.001	***
HbA1c (%)	4.83 ± 0.29	5.28 ± 0.63	2.902	0.006	**
FINS (μU/mL)	6.38 ± 4.49	10.87 ± 6.27	2.606	0.013	*
UA (mmol/L)	333.90 ± 100.23	353.57 ± 118.89	0.566	0.575	n.s
TG (mmol/L)	1.99 ± 1.80	1.96 ± 0.87	0.070	0.947	n.s
TC (mmol/L)	4.32 ± 0.71	4.96 ± 1.24	2.003	0.052	n.s
HDL-C (mmol/L)	1.25 ± 0.39	1.28 ± 0.37	0.250	0.804	n.s
LDL-C (mmol/L)	2.86 ± 0.69	3.23 ± 0.97	1.390	0.173	n.s
HOMA-IR	1.55 ± 1.14	2.95 ± 1.79	0.950	0.005	**
HOMA-IS	0.96 ± 0.56	0.53 ± 0.40	2.794	0.008	**
HOMA-β (%)	66.86 ± 40.66	86.88 ± 53.56	1.331	0.191	n.s

*SBP* systolic blood pressure, *DBP* diastolic blood pressure, *FBG* fasting blood glucose, *2-h PG* 2-hour post-load blood glucose, *HbA1c* glycosylated hemoglobin, *FINS* fasting insulin, *UA* uric acid, *TG* triglyceride, *TC* total cholesterol, *HDL-C* high-density lipoprotein cholesterol, *LDL-C* low-density lipoprotein cholesterol, *HOMA-IR* homeostatic model assessment for insulin resistance, *HOMA-IS* homeostatic model assessment for insulin sensibility, *HOMA-β* homeostatic model assessment of β-cell function. *n.s.* not significant, *p < 0.05, **p < 0.01, ***p < 0.001. The Mann–Whitney U test was used to test for significant differences between the groups, data are n (%) or mean ± SD

### Metabolomics analysis of PreDM with PXSK syndrome

**Fig. 1:**
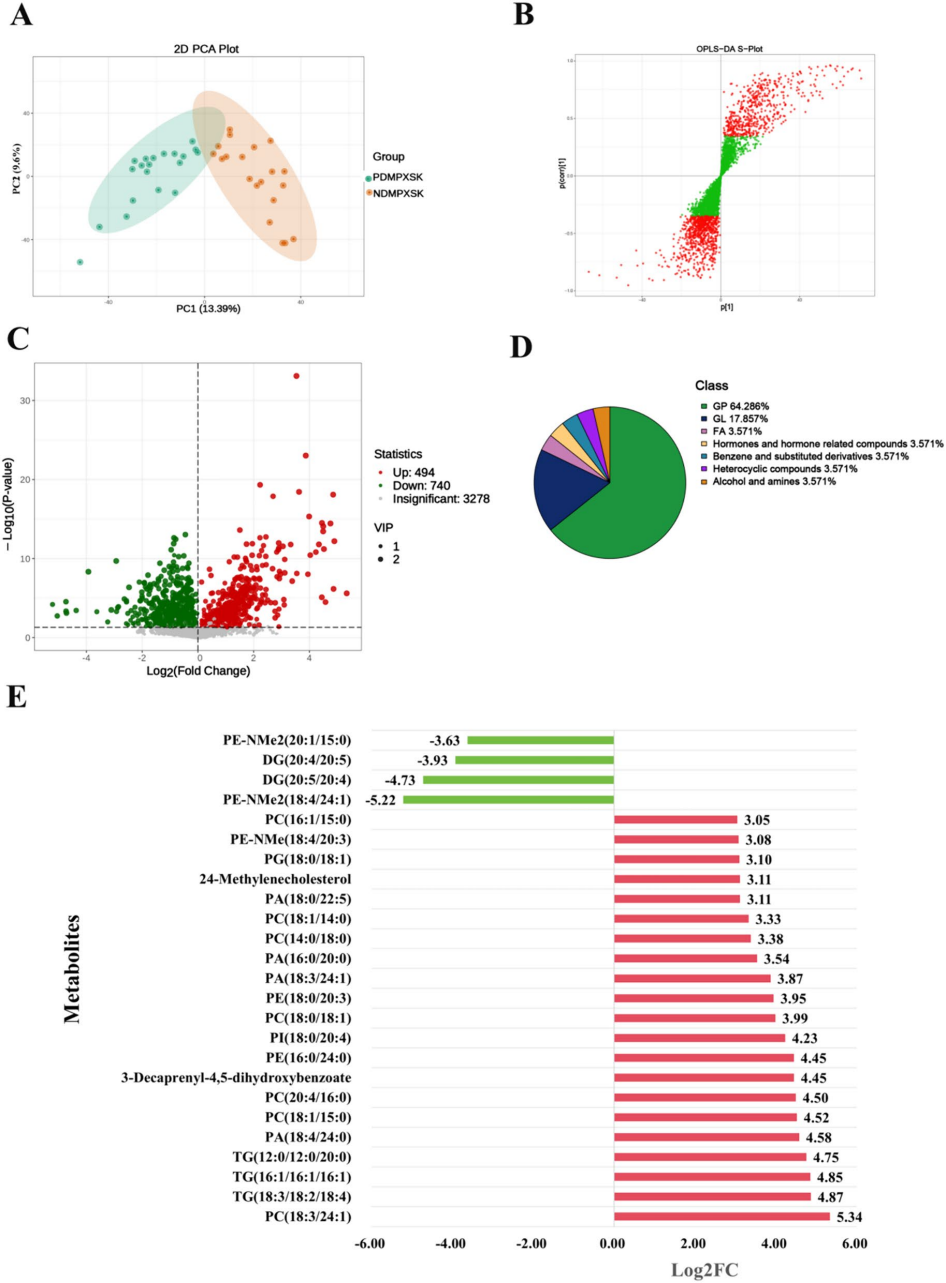
Metabolomics analysis of PreDM with PXSK syndrome. **A** PCA plot. **B** S-plots. In OPLS-DA, the closer the metabolic features are to the upper right and lower left corners, the more significant their differences are. The red dots indicate VIP ≥ 1, and the green dots indicate VIP < 1. **C** Volcano plots. In the Volcano plot, the red dots indicate metabolic features Log2FC > 0 were up-regulation, and green dots indicate metabolic features Log2FC < 0 were down-regulation. **D** Class classification chart for potential plasma markers. **E** Column chart of fold change of potential plasma markers in patients with the PDMPXSK. Using Log2FC > 3 and Log2FC < − 3 as the screening criteria, the red bars indicate these metabolites were increased, the green bars indicate these metabolites were decreased, the X axis was log2FC, Y axis was metabolite

The PCA plot showed that the metabolomics profiles of the plasma could be well separated between the NDMPXSK and PDMPXSK groups (R2X = 0.262, R2Y = 0.864, Q2 = 0.778) (Fig. 1A). A total of 1396 metabolic features were selected based on the VIP score of the OPLS-DA model (VIP > 1) (Fig. 1B). The Volcano plot showed that a total of 1466 features were significantly altered based on *P*-value < 0.05, among which 1234 differential metabolites met both VIP > 1 and *P*-value < 0.05 (740 metabolites were significantly down-regulated, while 494 metabolites were up-regulated.) (Fig. 1C). The 1234 differential metabolites that met the screening criteria were categorized, with a high percentage being benzene and substituted derivatives, glycerophospholipids, amino acid and its metabolites (Fig. S1A).

Subsequently, we applied an additional filter based on the fold-change of the metabolite to screen for potential plasma markers associated with PreDM in patients with PXSK syndrome. A total of 25 candidate metabolites were obtained using Log2FC > 3 and Log2FC < -3 as the screening criteria. The classification of these metabolites revealed that most of the metabolites belonged to glycerophospholipids (GP) and glycerolipids (GL) (Fig. 1D). In the PDMPXSK patients, 21 metabolites were significantly increased, while 4 metabolites were significantly decreased (Fig. 1E and Fig. S1B). Sixteen GPs (7 phosphatidylcholines (PCs), 4 phosphatidic acids (PAs), 2 phosphatidylethanolamines (PEs), 1 N-dimethyl-phosphatidylethanolamines (PE-NMe) 1 phosphatidylglycerol (PG), and 1 Phosphatidylinositol (PI)), three GLs (3 triglycerides (TGs)), 1 benzene and substituted derivatives (3-Decaprenyl-4,5-dihydroxybenzoate) and 1 hormones and hormone related compounds (24-Methylenecholesterol) were significantly elevated in the PDMPXSK group compared with the normal blood glucose populations (NDMPXSK group). At the same time, 2 N,N-dimethyl-phosphatidylethanolamines (PE-NMe2) and 2 Diacylglycerols (DGs) were significantly decreased in PDMPXSK vs NDMPXSK.

### Metabolomics analysis of PreDM with SRYP syndrome

**Fig. 2:**
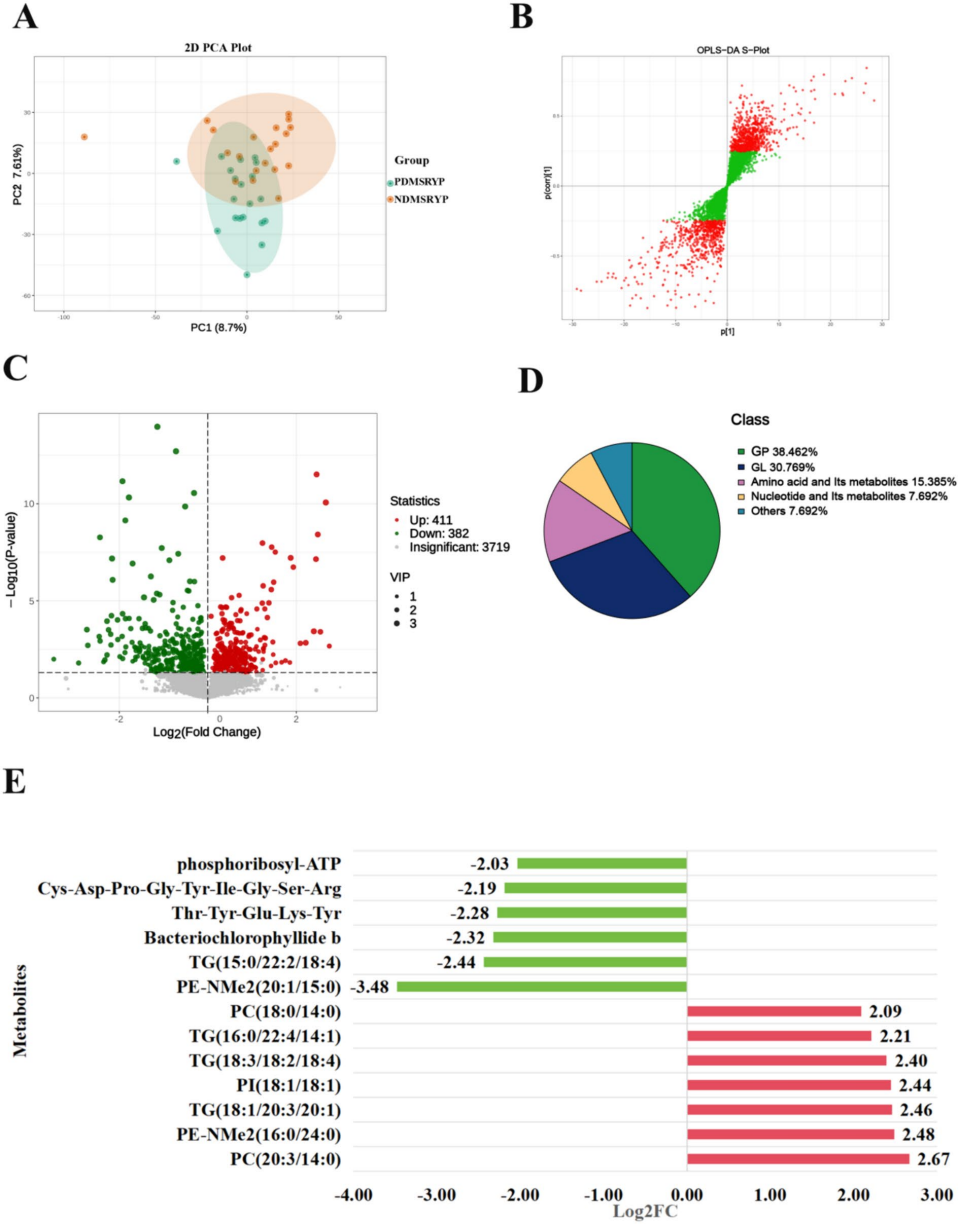
Metabolomics analysis of PreDM with SRYP syndrome. **A** PCA plot. **B** S-plots. In OPLS-DA, the closer the metabolic features are to the upper right and lower left corners, the more significant their differences are. The red dots indicate VIP ≥ 1, and the green dots indicate VIP < 1. **C** Volcano plots. In the Volcano plot, the red dots indicate metabolic features Log2FC > 0 were up-regulation, and green dots indicate metabolic features Log2FC < 0 were down-regulation. **D** Class classification chart for potential plasma markers. **E** Column chart of fold change of potential plasma markers in patients with the PDMSRYP. Using Log2FC > 2 and Log2FC < − 2 as the screening criteria, the red bars indicate metabolites that were increased, the green bars indicate metabolites that were decreased, the X axis represents log2FC, the Y axis represents metabolite

The PCA plots showed that the metabolomics profiles of the plasma were only moderately separated between the NDMSRYP and the PDMSRYP groups (R2X = 0.165, R2Y = 0.869, Q2 = 0.663) (Fig. 2A). A total of 1398 metabolic features were selected based on the VIP score of the OPLS-DA model (VIP > 1) (Fig. 2B). The Volcano plot showed that a total of 811 differential metabolites were screened based on *P*-value < 0.05, among which 793 differential metabolites met both VIP > 1 and *P*-value < 0.05 (382 metabolites were significantly down-regulated, while 411 metabolites were up-regulated.) (Fig. 2C). The 793 differential metabolites that met the screening criteria were categorized, with a high percentage being amino acid and its metabolites, benzene and substituted derivatives, heterocyclic compounds (Fig. S2A).

Subsequently, we applied an additional filter based on the fold-change of the metabolite to screen for potential plasma markers associated with PreDM in patients with SRYP syndrome. A total of 13 candidate metabolites were obtained by using Log2FC > 2 and Log2FC < − 2 as the screening criteria. Classification of these metabolites revealed that most of the metabolites belonged to glycerophospholipids (GP), glycerolipids (GL), and amino acid and its metabolites (Fig. 2D). In the PDMSRYP patients, 7 metabolites were significantly increased and 6 metabolites were significantly decreased (Fig. 2E and Fig. S2B). Among them, we found that 4 GPs (2 PCs, 1 PE-NMe2 and 1 PI) and 3 GLs (3 TGs) were significantly increased in the PDMSRYP compared with the normal blood glucose populations (NDMSRYP group), while 1 PE-NMe2, 1 TG, and 2 amino acid and its metabolites, 1 nucleotide and its metabolites (phosphoribosyl-ATP) and 1 Others (Bacteriochlorophyllide b) were significantly decreased.

### Differential pathway analyses

**Fig. 3:**
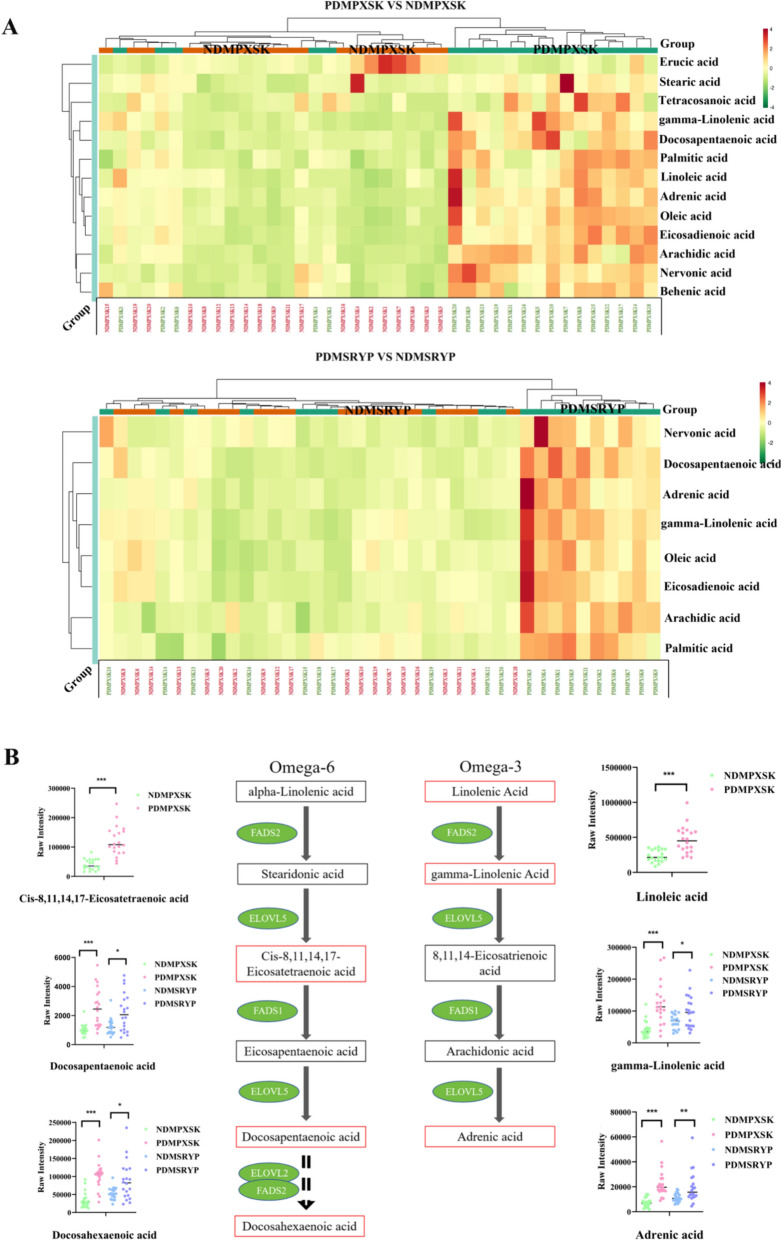
Plasma metabolite pathway enrichment in the PDMPXSK group and the PDMSRYP group compared to their respective normoglycemic controls. **A** Heat map of metabolites on the biosynthesis of unsaturated fatty acids. The picture above showed the metabolites on the pathway when the PDMPXSK group was compared with the NDMPXSK group, while the picture below showed the metabolites on the pathway when the PDMSRYP group was compared with the NDMSRYP group. **B** Pathway of metabolites on the alpha-linolenic acid and linoleic acid metabolism. The red box indicates that this metabolite was up-regulated in the pathway. Whereas the black box signifies that this metabolite was unchanged or undetectable in the pathway

The KEGG pathway analysis of the 1234 differential metabolites from the PDMPXSK vs NDMPXSK patients, showed 119 pathways were enriched, among which 23 enriched pathways had a *p*-value < 0.05. These metabolites were significantly enriched in "Glycerophospholipid metabolism", "Retrograde endocannabinoid signaling", and "Biosynthesis of unsaturated fatty acids" (Fig. S3A). Additionally, the 793 differential metabolites between the PDMSRYP vs NDMSRYP were enriched in 121 pathways, among which 8 enriched pathways had a *p*-value < 0.05. These metabolites were significantly enriched in the "Biosynthesis of unsaturated fatty acids", "Glycosylphosphatidylinositol (GPI)—anchor biosynthesis", and "Autophagy – animal" (Fig. S3B). Interestingly, the "biosynthesis of unsaturated fatty acids" pathway was significantly enriched in both syndromes, and all the differential metabolites enriched in this pathway were fatty acids, with almost all metabolites being elevated in the PDMPXSK group compared to NDMPXSK group and PDMSRYP group compared to NDMSRYP group, such as palmitic acid (FA 16:0), oleic acid (FA 18:1), gamma-linolenic acid (FA 18:3). Although the fold change of individual metabolites involved in this biological process was not sufficiently large to be considered a potential plasma biomarker, the results of pathway analysis considering the metabolome as a whole suggested that PreDM was profoundly associated with perturbed fatty acid metabolism (Fig. 3A).

The Small Molecule Pathway Database (SMPDB) pathway analysis of 1234 metabolites from PXSK patients has identified 365 enriched pathways, among which 9 enriched pathways had a *p*-value < 0.05 (Fig. S3D). Similarly, the SMPDB pathway analysis of 791 metabolites from SRYP patients revealed 333 pathways, with 2 of those enriched pathways had a *p*-value < 0.05 (Fig. S3E). Notably, alpha-linolenic acid and linoleic acid metabolism were significantly enriched in both syndromes. Docosapentaenoic acid (DPA), docosahexaenoic acid (DHA), gamma-linolenic acid and adrenic acid (the MS/MS spectra shown in Fig. S6A) on this pathway were increased both in the PDMPXSK and PDMSRYP groups compared to their respective normoglycemic controls. Cis-8,11,14,17-Eicosatetraenoic acid (the MS/MS spectra shown in Fig. S6B) acid and linolenic acid were increased in the PDMPXSK group compared to the NDMPXSK group and were not detected in the PDMSRYP group compared to the NDMSRYP group. Again, although the overall fold change in these metabolites was not high enough to be used as a clinical indicator, this result suggested that plasma metabolites of α-linolenic acid and linoleic acid have been disturbed in PreDM patients (Fig. 3B).

### The common metabolite markers of PreDM with PXSK syndrome and PreDM with SRYP syndrome

**Fig. 4:**
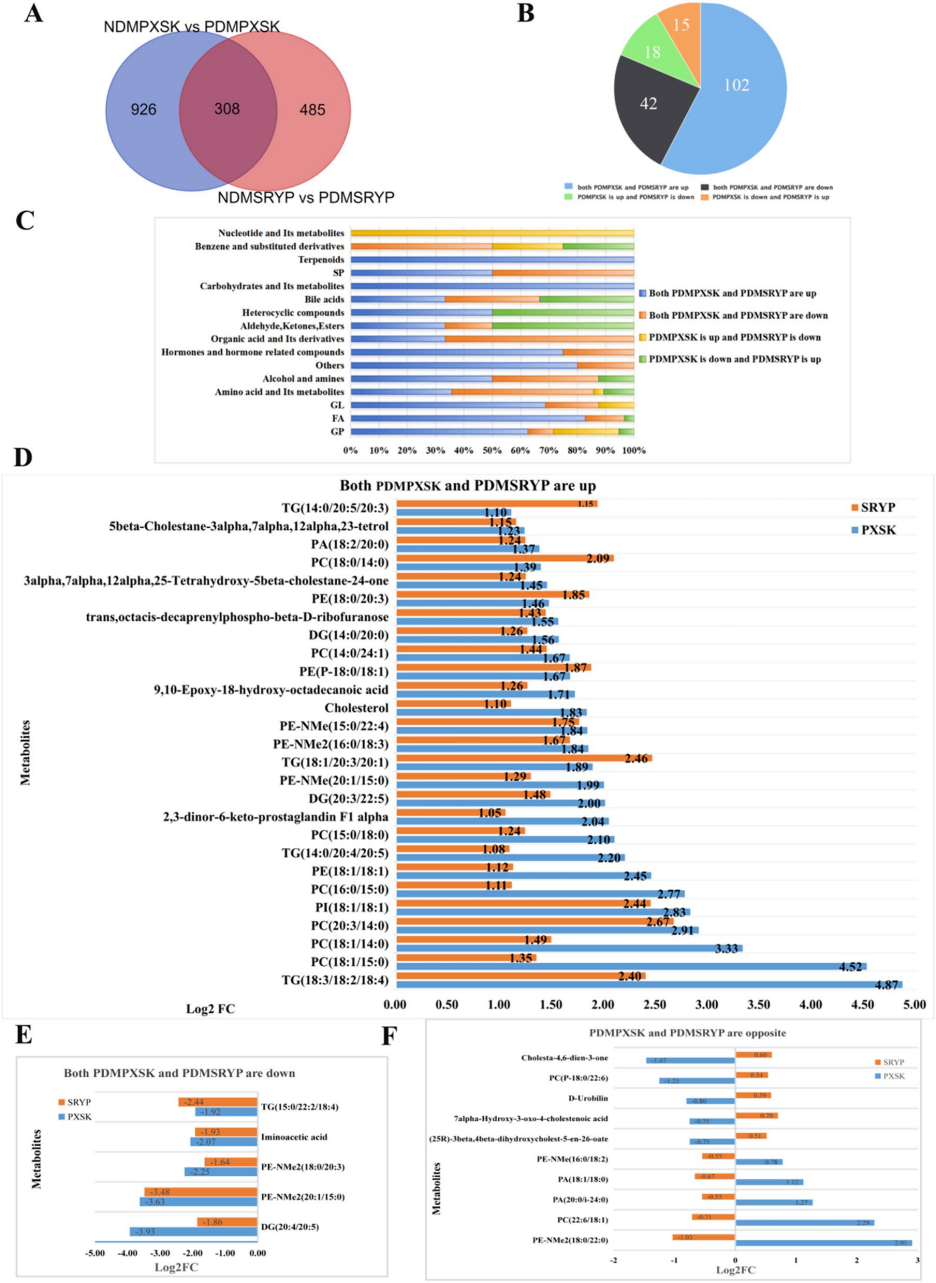
The common metabolite makers of PreDM with PXSK syndrome and PreDM with SRYP syndrome. **A** Venn diagram displaying the common differential metabolites when the PXSK group was compared with the SRYP group. **B** Class classification chart for tentatively annotated as endogenous metabolites. The blue color indicates the endogenous metabolites both in the PDMPXSK group vs NDMPXSK group and the PDMSRYP group vs NDMSRYP group were increased. The black color indicates the endogenous metabolites both in the PDMPXSK group vs NDMPXSK and the PDMSRYP group vs NDMSRYP group were decreased. The green color indicates that endogenous metabolites were increased in the PDMPXSK group vs NDMPXSK but decreased in the PDMSRYP group vs NDMSRYP group. The orange color indicates that endogenous metabolites were decreased in the PDMPXSK group vs NDMPXSK group but increased in the PDMSRYP group vs NDMSRYP group. **C** Class classification chart of common endogenous metabolites. The X axis is the percentage of a metabolite category in total metabolites, and the Y axis is the metabolite category, with closer to the bottom indicating a higher number of metabolites, and closer to the top a lower number of metabolites. **D** Potential elevated plasma markers in PreDM patients. Using Log2FC > 1 as the screening criteria, the blue bar was the PXSK group, the orange bar was the SRYP group, the X axis was log2FC, Y axis was metabolite. **E** Potentially reduced plasma markers in PreDM patients. Using Log2FC < − 1 as the screening criteria, the blue bar was the PXSK group, the orange bar was the SRYP group, the X axis was log2FC, Y axis was metabolite. **F** Potential plasma markers with opposite changes in the PDMPXSK group vs NDMPXSK group and the PDMSRYP group vs NDMSRYP group. Using Log2FC > 0.5 and Log2FC < − 0.5 as the screening criteria, the blue bar was the PXSK group, the orange bar was the SRYP group, the X axis was log2FC, Y axis was metabolite

In order to identify PreDM-associated metabolites that are common to PXSK and SYRP syndromes, Venn diagram analysis was employed. A total of 308 differential metabolites were found to be common, of which 177 were tentatively annotated as endogenous metabolites (Fig. 4A). Classification of these 177 metabolites showed that these common metabolites mainly encompassed glycerophospholipids (GP), fatty acids (FA), amino acid and its metabolites, glycerolipids (GL) (Fig. S4A).

Next, we examined and further categorized the altered metabolites based on the direction of changes. One hundred and two endogenous metabolites were found to be elevated both in the PDMPXSK group and the PDMSRYP group compared to their respective normoglycemic controls, encompassing glycerophospholipids, fatty acids, glycerolipids, amino acids and its metabolites. On the other hand, forty-two endogenous metabolites were found to be decreased both in the PDMPXSK group vs NDMPXSK group and in the PDMSRYP group vs NDMSRYP group, and classification of these commonly decreased metabolites revealed a high percentage of amino acid and its metabolites, organic acid and its derivatives, glycerophospholipids, and fatty acids. Finally, we found 33 endogenous metabolites showing an opposite direction of change, with 18 metabolites showing an increased in the PDMPXSK group vs NDMPXSK group but decreased in the PDMSRYP group vs NDMSRYP group, whereas 15 metabolites were decreased in the PDMPXSK group vs NDMPXSK group but increased in the PDMSRYP group vs NDMSRYP group. These metabolites mainly belong to glycerophospholipids, amino acid and its metabolites, and aldehyde, ketones, esters (Fig. 4B and C).

To further narrow down the number of potential markers for PreDM regardless the TCM syndrome, an additional filter based on fold change was applied. Among the 102 endogenous metabolites that were simultaneously elevated, using Log2FC > 1 as the screening condition, a total of 27 possible metabolites were obtained (Fig. 4D), of which 15 metabolites belonged to GPs (7 PCs, 2 PEs, 2 PE-NMe, 1 PE-NMe2, 1 Ether-linked PE, 1 PI, 1 PA), 6 metabolites belonged to GLs (4 TGs, 2 DGs), 2 metabolites belonged to hormones and hormone related compounds (2,3-dinor-6-keto-prostaglandin F1 alpha, cholesterol (the MS/MS spectra shown in Fig. S6C)), 1 metabolite belonged to fatty acid (9,10-Epoxy-18-hydroxy-octadecanoic acid), 1 belonged to alcohol and amines (5beta-Cholestane-3alpha,7alpha,12alpha,23-tetrol) and 1 belonged to aldehyde, ketones, esters (3alpha,7alpha,12alpha,25-Tetrahydroxy-5beta-cholestane-24-one) (Fig. S4B). Among the 42 endogenous metabolites were reduced in PreDM with either syndrome, a total of 5 possible metabolites were obtained using Log2FC < − 1 as the screening condition (Fig. 4E), of which 2 metabolites belonged to GPs (2 PE-NMe2), 2 metabolites belonged to GLs (1 TG, 1 DG), and 1 belonged to organic acid and its derivatives (Iminoacetic acid) (Fig. S5A). Among the 33 endogenous metabolites with opposite changes, a total of 10 possible metabolites were obtained by Log2FC > 0.5 or Log2FC < − 0.5 (Fig. 4F), of which 6 metabolites were GPs (Fig. S5B). 2 PAs, 1 PE-NMe2, 1 PE-NMe, and 1 PC were elevated in the PDMPXSK but were decreased in the PDMSRYP compared to their respective normoglycemic controls, while 1 Ether-linked PC was decreased in the PDMPXSK vs NDMPXSK but was increased in the PDMSRYP vs NDMSRYP. (Fig. S5B). Also, we also found that cholesterol and its related metabolites (i.e. (25R)-3beta, 4beta-dihydroxycholest-5-en-26-oate, Cholesta-4,6-dien-3-one, 7alpha-Hydroxy-3-oxo-4-cholestenoic acid (the MS/MS spectra shown in Fig. S6D)) and heterocyclic compounds (D-Urobilin) were reduced in the PDMPXSK vs the NDMPXSK group, but elevated in the PDMSRYP group vs the NDMSRYP group. The above results suggest that metabolites of glycerophospholipids and glycerolipids have great potential to be used as plasma markers in clinical PreDM patients.

### Association between metabolite markers and clinical measurements

To further demonstrate the implication of the altered plasma metabolites, Pearson correlation analysis was employed to identify potential links between metabolites and 8 blood biochemical indices, including 4 glycaemia-related (FBG, 2-h PG, HbA1c, FINS) and 4 lipid profile-related (TG, TC, HDL-C, LDL-C) (Table S2B). Here we focused on metabolites commonly increased (Fig. 4D), or decreased (Fig. 4E) in the comparison of both PDMPXSK vs NDMPXSK and PDMSRYP vs NDMSRYP (Some MS/MS fragments of candidate metabolite markers are shown in Table S4). Regarding the glycaemia-related indicators, FBG was positively correlated with 3α,7α,12α,25-Tetrahydroxy-5β-cholestane-24-one (r = 0.324, *p* = 0.0412) and PA (18:2/20:0) (r = 0.319, *p* = 0.0449). FINS was positively correlated with 3α,7α,12α,25-Tetrahydroxy-5β-cholestane-24-one (r = 0.364, *p* = 0.0208), 1,2-Dioleoylphosphatidylethanolamine (r = 0.356, *p* = 0.0242), PE-NMe (20:1/15:0) (r = 0.333, *p* = 0.0358), PE-NMe2 (16:0/18:3) (r = 0.354, *p* = 0.0250), PE-NMe (15:0/22:4) (r = 0.520, *p* = 0.0006), and negatively correlated with PC (18:1/14:0) (r = − 0.343, *p* = 0.0303). No significant correlation could be identified for HbA1c or 2-h PG.

**Fig. 5:**
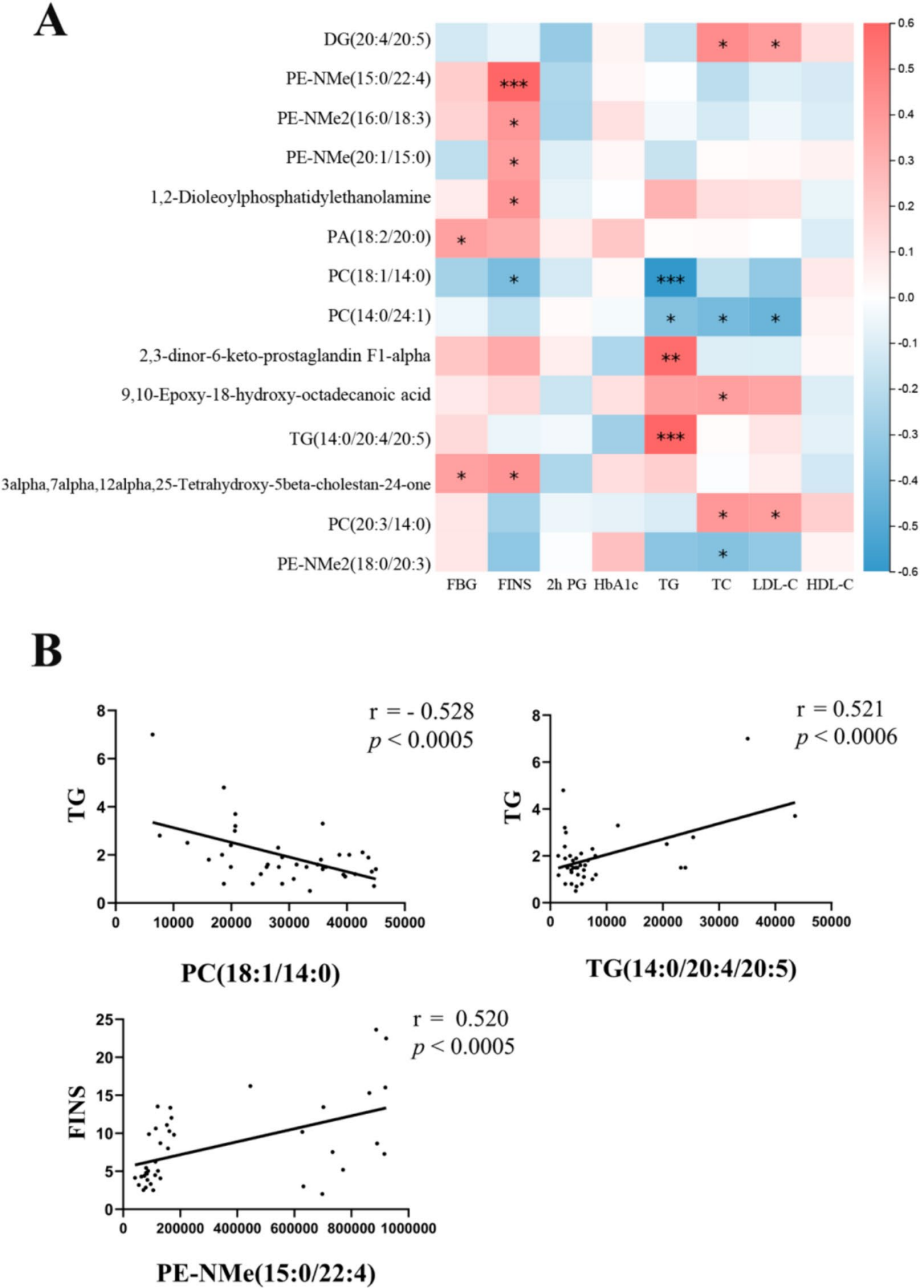
The potential plasma markers in clinically PreDM patients. **A** The correlation of change between the metabolites both in PDMPXSK group vs NDMPXSK group and in PDMSRYP group vs NDMSRYP group with clinical variables. *P*-values < 0.05 in the comparison of the two groups are marked with a box. **B** The correlation plots for metabolites with the | r |> 0.5 and* p* < 0.05

Regarding the clinical lipid profile-related indicators, TG was negatively correlated with PC (18:1/14:0) (r = − 0.528, *p* = 0.0005) and PC (14:0/24:1) (r = − 0.319, *p* = 0.0448), and positively correlated with TG (14:0/20:4/20:5) (r = 0.521, *p* = 0.0006) and 2,3-dinor-6-keto-prostaglandin F1 α (r = 0.497, *p* = 0.0011). TC was negatively correlated with PC (14:0/24:1) (r = − 0.352, *p* = 0.0257) and PE-NMe2 (18:0/20:3) (r = − 0.317, *p* = 0.0466), and positively correlated with DG (20:4/20:5) (r = 0.391, *p* = 0.0127), 9,10-Epoxy-18-hydroxy-octadecanoic acid (r = 0.327, *p* = 0.0397) and PC (20:3/14:0) (r = 0.350, *p* = 0.0268). LDL-C was negatively correlated with PC (14:0/24:1) (r = − 0.384, *p* = 0.0143), positively correlated with PC (20:3/14:0) (r = 0.334, *p* = 0.0354) and DG (20:4/20:5) (r = 0.336, *p* = 0.0338). No significant correlation was observed for HDL-C. The corresponding results are shown in Fig. 5 and Table S2A.

## Discussion

As an asymptomatic period, PreDM patients usually present no obvious physical discomfort, yet from the point of view of TCM constitution, qi stagnation constitution, and spleen deficiency syndrome may already have existed [11]. These patients could miss the optimal window for managing their condition with Chinese medicine since the progression is insidious and difficult to detect. According to TCMs, PreDM is often associated with the syndrome of spleen deficiency with dampness encumbrance [20] and the syndrome of dampness-heat in the spleen [21]. If these patients receive attention and intervention at an early stage, the progression of the disease can be largely prevented. In addition, early identification of individuals at risk of diabetes is a cost-effective strategy to reduce the incidence of diabetes [22]. Consequently, the role of Chinese medicine in the prevention and treatment of PreDM deserves further in-depth research. Currently, the identification of these patterns primarily depends on the clinical evaluation by TCM physicians, molecular biomarkers are not yet known. Metabolomics has been widely used to discover biomarkers for the diagnosis, treatment and prevention of diseases, and has become a powerful tool to explore the scientific fundamental underlying TCM theories [23–25]. Small molecule biomarkers identified by metabolomics can provide insight into metabolic perturbations contributing to the development of PreDM and help identify at-risk individuals. At present, most metabolomics studies focus on biomarker screening for PreDM without distinguishing different TCM syndromes of PreDM. Herein, we identified biomarkers for PreDM, taking into account the similarities and differences between two TCM syndromes that are often associated with the TCM-based diagnosis of PreDM. Although PreDM patients with PXSK or SRYP were characterized by different clinical profiles and metabolomics signatures, our study was also able to identify common metabolites and perturbed metabolic pathways significantly associated with PreDM shared by the two different TCM syndromes.

Under the syndrome of SRYP, we found that the BMI of the PreDM group was significantly higher than that of the normal blood glucose group. Overweight and obesity have been a longstanding, well-established risk factor for the development of T2DM. An excess accumulation of body fat exceeding the storage capacity of adipose tissue can increase the level of circulating free fatty acid (FFA) and contribute to lipotoxicity and the development of insulin resistance (IR) [26, 27]. In line with this, our data also revealed significantly reduced insulin sensitivity (HOMA-IS) in the PreDM patients with SRYP, whereas no difference was observed in those with PXSK syndrome, and there was a negative correlation between HOMA-IS and BMI. The development of T2DM can be generally categorized into obesity-related and obesity-independent types. The co-occurrence of elevated BMI and dysglycemia observed in PDMSRYP patients resembled the obesity-related T2DM pattern. Since obesity was only present in PDMSRYP and not in PDMPXSK, our findings also suggest that the SRYP population may be more susceptible to the development of obesity-related dysglycemia, whereas dysglycemia in the PXSK group is mainly caused by pathogenic mechanisms unrelated to body weight [27, 28]. Consequently, we proposed that weight control is an important approach for the prevention of SRYP population to enter the early stage of dysglycaemia as well as the further progression of PreDM towards T2DM.

In adherence to the *Guideline for the prevention and treatment of type 2 diabetes mellitus in China (2020 edition)* where FBG, 2-h PG and HbA1c are used as diagnostic basis for diabetes [1], we found that PreDM patients with either syndrome exhibited higher levels of 2-h PG and HbA1c levels compared to their normal blood glucose controls. Intriguingly, PreDM patients with SRYP also had elevated levels of FBG and FINS, suggesting a different mode of glycemic abnormality between the two TCM syndromes, with PDMPXSK being mainly associated with IGT, whereas PDMSRYP is predominantly characterized by the presence of both IFG and IGT (Table S3). Accordingly, IGT typically arises from peripheral IR or a reduction in second-phase insulin release, whereas IFG is mainly caused by impaired insulin suppression of basal glucose output due to hepatic IR or decreased basal insulin secretion [29].

At the global level of metabolomics profiling, we reported the PreDM-associated signature characteristic of each TCM syndrome. PDMPXSK patients showed elevated levels of several GPs (7 PCs, 4 PAs, 2 PEs, 1 PE-NMe, 1 PG and 1 PI) and 3 GLs (3 TGs), as well as decreased levels of 2 DGs and 2 PE-NMe2 compared to NDMPXSK. In PDMSRYP, we found elevation in the levels of 4 GPs (2 PCs, 1 PE-NMe2 and 1 PI) and 3 TGs, while 1 PE-NMe2 and 1 TG species as well as 2 amino acid and its metabolites were significantly decreased compared to NDMSRYP. Although the constituent of the panel of discriminatory metabolites associated with PreDM was quite different between PXSK and SRYP at the level of individual molecule, many of them belong to the class of glycerophospholipids and glycerolipids. These findings suggested that perturbation in the levels of a number of lipid species was a consistent trait during early stage of dysglycaemia across the two TCM syndromes examined in the present study. In parallel, enrichment analysis based on the individual set of discriminatory metabolites obtained from the PXSK cohort and the SRYP cohort both revealed fatty acid metabolism as one of the most important altered pathways in PreDM, further supporting the engagement of perturbed lipid metabolism during early dysglycaemia. Meanwhile, these "syndrome-specific" metabolites may serve as additional candidate markers to facilitate PreDM diagnosis in sub-population identified as PXSK or SRYP, which of note requires further validation in a separate cohort.

In an attempt to discover common PreDM biomarkers shared by the two TCM syndromes, we applied a less strict selection criterion for biomarker screening. Interestingly, we found increased levels of cholesterol along with two steroid derivatives involved in bile acid biosynthesis, among which the metabolite 5beta-Cholestane-3alpha,7alpha,12alpha,26-tetrol was also positively correlated with two glycaemia-related clinical indicators, FINS and FBG. Although bile acid (BA) is primarily metabolized in the enterohepatic system and mainly plays a biological function in facilitating fat absorption, it is increasingly recognized that BA can also modulate metabolism and biological processes in distal organ sites via signaling transduction of the BA receptors such as FXR and TGR5. T2DM has been found to be associated with an increased BA pool size, and insulin treatment could reduce the level of BA [30], possibly via an inhibition of BA biosynthesis as in vivo evidence showed that insulin suppressed the expression of the rate-limiting enzymes involved in BA biosynthesis [31]. Interestingly, BA chelating agent was found to improve blood glucose levels in T2DM patients [32]. However, later studies suggested that it was the composition of BA, rather than the total BA pool size, associated with T2DM, although it remains inconclusive the detailed pattern of changes [33]. Without the observation of changes in the levels of individual BA composition between PreDM and normal blood glucose controls in our study, it was hard to comment beyond this point, yet our findings reinforced a tight association between disruption in BA homeostasis and the development of dysglycaemia. Future targeted studies focusing on BA profiling may help to dissect this relationship. As for the prognostic value of BA in T2DM risk prediction, although we and others demonstrated there were some associations between the levels of BA-related metabolites and clinical glycaemia-related indicators, fasting plasma BA failed to predict new-onset diabetes in patients with IFG [34]. As such, the causal link between altered BA homeostasis and worsening of glycaemic control also warrants future mechanistic study.

We have also identified a collection of GLs (4 TGs, 2 DGs) and GPs (7 PCs, 2 PEs, 2 PE-NMe, 1 ether-linked PE, 1 PE-NMe2, 1 PI, 1 PA) to be increased in PreDM, whereas 1 TG, 1 DG and 2 PE-NMe2 species were decreased. Dysregulation of lipid metabolism has been tightly associated with the pathogenesis of T2DM and is suggested to be an early event before the onset of dysglycaemia [35]. Meikle et al. have emphasized a tight association between the plasma levels of several phospholipids and triglycerides and incident PreDM/T2DM [36]. In a previous study characterizing the metabolomic signature associated with PreDM in a mixed ethnic cohort, FPG level in Asian Chinese was found to be profoundly correlated with the levels of a number of TGs, DGs, PCs and PEs as well as ceramides [37]. Our present study was able to recapitulate some of these findings including elevations in the levels of several TGs and PCs level in PreDM patients irrespective of their TCM syndromes, highlighting changes in the levels of these lipid classes could be a well conserved metabolic trait during early dysglycaemia. In addition, higher basal levels of TGs, DGs, PCs and PEs were associated with an increased risk of T2DM in a longitudinal study [38], suggesting a global change in these lipid species does not only serve as molecular markers for PreDM but may also be causally linked to pathogenesis of T2DM. PCs are the most abundant lipid species of plasma membrane, but are also enriched in lipoprotein particles along with TGs and cholesterol. A concomitant elevation in the level of TGs and PCs may be related to hypertriglyceridemia or increased production of very-low-density lipoprotein (VLDL) which contains a TG-rich core encapsulated in PC layer. Increased TG may be also related to regulation of macrophage and inflammation [39]. PE is a key molecule in the synthesis of GPI [40]. The GPI binds and tethers some proteins to the outer leaflet of the cytoplasmic membrane to perform their biological functions, such as GPLD1 and GPIHBP1, which are both implicated in the development of T2DM [41, 42]. We also observed a positive correlation between PA (18:2/20:0) and FBG in PreDM patients, these findings were consistent with previous studies [43].

We have intriguingly observed a positive correlation of some N-methylated PEs (i.e., PE-NMe(20:1/15:0), PE-NMe2(16:0/18:3), PE-NMe(15:0/22:4)) with FINS. However, no unified direction of changes in this lipid class could be concluded when comparing between PreDM and normal blood glucose (i.e. some species were up and some were down). PE-NMe and PE-NMe2 are intermediates of the methylation pathway for PE-to-PC conversion by phosphatidylethanolamine N-methyltransferase (PEMT). Although the contribution of this pathway to the total PC pool is minor as opposed to the Kennedy pathway, PC derived from the PE methylation was found to be critical for maintaining hepatic VLDL production and secretion, and mice deficient in *Pemt* developed massive steatosis (Non-alcoholic fatty liver disease, NAFLD) and eventually progressed to non-alcoholic steatohepatitis (NASH) [44]. Interestingly, a V175M loss-of-function mutation in the PEMT gene was reported in human subjects and tightly associated with NAFLD and NASH in the East Asian population [45, 46]. Hepatic PEMT expression was also found to inversely correlate with NAFLD severity [47]. Noteworthy, these liver diseases are tightly linked to hepatic IR and pose a huge risk to T2DM development. A more recent Genome-Wide Association Studies (GWAS) study examined the association between PEMT in different sites and metabolic traits, and intriguingly identified the visceral PEMT to be significantly correlated with both T2DM and NAFLD [48]. As a result, the inconclusive pattern of changes in the level of different N-methylated PE species might be due to the fact that the circulating level of this lipid class as measured in the present study was affected by multiple tissue sites. Nonetheless, our study shed new light on the involvement of impaired methylation of PE during the development of dysglycaemia.

Lipids such as fatty acids (FA), fatty acid esters of hydroxy fatty acids, diacylglycerols and ceramides, can modulate the insulin signaling pathway. Besides, lipids as metabolic substrates can fine-tune the balance between lipid and glucose metabolism as well as affecting hepatic glucose production, which in turn regulates insulin sensitivity [49]. Interestingly, metabolites associated with PreDM patients PXSK or SRYP were both found to be enriched in the KEGG pathway of biosynthesis of unsaturated fatty acids, a similar finding was drawn using an alternative database (SMPDB) for pathway enrichment analysis. The metabolites enriched in this pathway were mostly unsaturated fatty acids (USFA), including gamma-linolenic acid, adrenic acid, DPA, and DHA, all of which were found to be increased in PreDM patients. A previous study has reported the majority of n-6 polyunsaturated fatty acids (PUFA) were associated with a higher risk of T2DM [50]. Increased levels of circulating FFA have been a hallmark feature of obesity-related insulin resistance. Increased FFA could be caused by obesity where there is an uncontrolled release of FFA into the circulation by the adipose tissue. The elevated circulating FFA can flux into non-adipose tissue such as the liver and muscle and cause ectopic fat deposition, worsening the insulin sensitivity in these metabolically active sites [51]. At the cellular level, PUFAs are important components of membrane phospholipids, and the relative content of PUFAs in the membrane phospholipids could affect the fluidity and permeability. However, how membrane dynamics as in part determined by the component of phospholipids as well as the PUFAs composition affects the localization and functioning of membrane proteins still remains largely unknown [52, 53]. In addition, PUFAs are prone to lipid oxidation and peroxidation, and hence a higher content of PUFAs could render cells greater susceptibility to oxidative and peroxidative damage. Elevated plasma FFA can lead to lipotoxicity [54], and lipotoxicity is associated with impaired pancreatic β-cell functioning, decreased systemic insulin sensitivity, and increased risk of cardiovascular and renal disorders [55, 56].

On the other hand, the plasma level of FFA is also largely affected by dietary influences. However, at present, findings on the effects of PUFAs on glucose metabolism and the prevention of T2DM remain controversial. Eichelmann et al. suggested that increasing the proportion of USFA substitutes in the diet in place of saturated fatty acids (SFA) is a potential way to prevent cardiovascular disease and T2DM [50]. SFAs reduce fat storage-inducing transmembrane protein 2 (FIT2) which leads to β cell dysfunction and death, leading to diabetes. It suggested that PreDM/T2DM patients should reduce their intake of SFA [57]. In contrast, Brown et al. found that increasing the intake of omega-3, omega-6, or total PUFA had little effect on the prevention and treatment of T2DM [58]. Without dietary records in the present study, we were unable to deduce whether the increases in a series of FFA were solely due to pathogenic process associated with dysglycaemia or long term dietary habits. Nonetheless, our findings reinforced an association between elevated circulating FFA and early stage of dysglycaemia.

### Limitations

Our study has some limitations: The lack of standardized clinical data indicators for the syndrome of spleen deficiency with dampness encumbrance and the syndrome of dampness-heat in the spleen, it is mainly assessed by TCM physicians on the basis of clinical syndromes, signs, tongue manifestation and pulse condition, which is somewhat subjective. Moreover, the metabolites of the syndrome of PXSK and SRYP in PreDM have not been reported in the literature, and it is impossible to combine the clinical syndrome indicators with the metabolites to find the markers for a specific pattern. In addition, the number of patients included in this study was small and all the included samples were used for the discovery of potential candidate markers. Further validation study needs to be conducted in the future.

## Conclusion

In conclusion, the present study for the first time employed untargeted metabolomics to study the metabolomics signature and identify potential metabolite markers associated with PreDM in patients with different TCM syndromes. The TCM syndromes investigated here included PXSK and SRYP as they were subtypes of "spleen-dampness" which was considered highly relevant to the development of dysglycaemia. Clinically, PDMPXSK patients were characterized by non-obesity and IGT compared with normal glucose controls with PXSK; while PDMSRYP patients were characterized by obesity and IGT + IFG compared with normal glucose controls with SRYP. Although the metabolomics signature characteristic for PreDM was quite different between PXSK and SRYP at a global scale, we were able to identify common metabolite markers associated with PreDM shared by the two TCM syndromes. In addition to a number of glycerolipids and glycerophospholipids that were already frequently reported to be associated with PreDM, the present study also observed significant associations between steroid derivatives involved in bile acid biosynthesis and clinical glycaemia-related indicators, as well as altered levels of N-methylated phosphatidylethanolamines in PreDM patients with either PXSK or SRYP. Our findings provided evidence for potential link between perturbed BA homeostasis and PreDM, as well as the possible involvement of dysregulated PE methylation pathway by PEMT during the early stage of dysglycaemia in patients with "spleen-dampness". Our study highlighted the suitability and robustness of the combined use of metabolomics and clinical study to discover biomarkers and explore scientific fundamentals underlying TCM theory.

## Supplementary Information

Supplementary Material 1

Supplementary Material 2

## Data Availability

The datasets used and/or analyzed among the current study are available from the corresponding author on reasonable request.

## Abbreviations

PreDM: Prediabetes mellitus
IFG: Impaired fasting glucose
IGT: Impaired glucose tolerance
T2DM: Type 2 diabetes mellitus
TCM: Traditional Chinese Medicine
PXSK: The syndrome of spleen deficiency with dampness encumbrance
SRYP: The syndrome of dampness-heat in the spleen
PDMPXSK: Patients with syndrome of spleen deficiency with dampness encumbrance and PreDM
PDMSRYP: Patients with syndrome of dampness-heat in the spleen and PreDM
NDMPXSK: Patients with syndrome of spleen deficiency with dampness encumbrance and normal blood glucose
NDMSRYP: Patients with syndrome of dampness-heat in the spleen and normal blood glucose
FBG: Fasting blood glucose
2-h PG: 2-hour post-load blood glucose
BMI: Body mass index
WHR: Waist-to-hip ratio
HbA1c: Glycosylated hemoglobin
FINS: Fasting insulin
TC: Total cholesterol
TG: Triglyceride
HDL-C: High-density lipoprotein cholesterol
LDL-C: Low-density lipoprotein cholesterol
UA: Uric acid
HOMA-IR: Homeostatic model assessment for insulin resistance
HOMA-IS: Homeostatic model assessment for insulin sensibility
HOMA-β: Homeostatic model assessment of β-cell function
PCA: Principal component analysis
VIP: Variable importance in projection
OPLS-DA: Orthogonal partial least-squares discriminant analysis
HMDB: Human metabolome database
KEGG: Kyoto Encyclopedia of Genes and Genomes
SMPDB: Small Molecule Pathway Database
GP: Glycerophospholipids
GL: Glycerol-esters
FA: Fatty acids
DG: Diacylglycerols
PC: Phosphatidyl choline
PE: Phosphatidyl ethanolamine
PE-NMe2: N,N-dimethyl-phosphatidylethanolamines
PE-NMe: N-dimethyl-phosphatidylethanolamines
PA: Phosphatidic acid
PG: Phosphatidylglycerol
PI: Phosphatidylinositol
CE: Ceramides
GPI: Glycosylphosphatidylinositol
BA: Bile acid
VLDL: Very-low density lipoprotein
PEMT: Phosphatidylethanolamine N-methyltransferase
NAFLD: Non-alcoholic fatty liver disease
NASH: Non-alcoholic steatohepatitis
IR: Insulin resistance
PUFA: Polyunsaturated fatty acids
USFA: Unsaturated fatty acids
SFA: Saturated fatty acids
FFA: Free fatty acids
DPA: Docosapentaenoic acid
DHA: Docosahexaenoic acid
