# Correction: Comprehensive biomarker analysis of metabolomics in different syndromes in traditional Chinese medical for prediabetes mellitus

**DOI:** 10.1186/s13020-025-01181-3

**Published:** 2025-12-22

**Authors:** Qin Lan, Xue Li, Jianhe Fang, Xinyu Yu, Zhanxuan E. Wu, Caiyun Yang, Hui Jian, Fei Li

**Affiliations:** 1 Jiangxi University of Traditional Chinese Medicine, Nanchang, 330004 China; 2 Department of Gastroenterology and Hepatology, Laboratory of Metabolomics and Drug-Induced Liver Injury, Frontiers Science Center for Disease-Related Molecular Network, and State Key Laboratory of Respiratory Health and Multimorbidity, West China Hospital, Sichuan University, Chengdu, 610041 Sichuan China; 3 Medical Ancient Literature Teaching and Research Office, Jiangxi University of Traditional Chinese Medicine, Nanchang, 330004 China; 4 Discipline of Chinese and Western Integrative Medicine, Jiangxi University of Chinese Medicine, Nanchang, 330004 China; 5 Outpatient Department, Hongdu Traditional Chinese Medicine Hospital Affiliated to Jiangxi University of Traditional Chinese Medicine, Nanchang, 330006 China; 6 Endocrinology Department II, Hongdu Traditional Chinese Medicine Hospital Affiliated to Jiangxi University of Traditional Chinese Medicine, Nanchang, 330006 China

**Correction: Chinese Medicine (2024) 19:114.** 10.1186/s13020-024-00983-1

Following publication of the original article [1], the authors found some mistakes in Tables 3 and 4.

This error is due to the wrong input of the raw data.

1. The HDL-C raw data of No.10 patient in PDMPXSK group is 1.02, not 102, which was used to calculate the numerical value of HDL-C in Table 3.
2. The insulin raw data of No. 2 and 7 in PDMSRYP is 14.41 and 18.02, not 4.41 and 8.02, respectively, which was used to calculate the numerical value of FINS, HOMA-IR, HOME-IS, HOMA-β in Table 4.

The correct Tables 3 and 4 have been provided in this Correction.

The incorrect Table 3 is:

**Table 3:** Comparison of the glycolipid-related indicators between the normal blood glucose and PreDM group among the PXSK patients

	NDMPXSK n = 20 (Male: 9 Female: 11)	PDMPXSK n = 20 (Male: 9 Female: 11)	t/z	*p*	
SBP (mmHg)	112.10 ± 7.13	122.30 ± 11.50	3.371	0.002	**
DBP (mmHg)	73.85 ± 6.08	77.85 ± 10.07	1.521	0.137	n.s.
FBG (mmol/L)	5.40 ± 0.39	5.82 ± 0.66	1.458	0.153	n.s.
2 h PG (mmol/L)	5.57 ± 0.92	8.88 ± 1.26	9.489	0.001	***
HbA1c (%)	4.76 ± 0.37	5.46 ± 0.49	5.100	0.001	***
FINS (μU/mL)	8.04 ± 5.58	6.60 ± 3.92	0.944	0.351	n.s.
UA (mmol/L)	363.90 ± 88.36	295.48 ± 104.47	2.236	0.031	*
TG (mmol/L)	1.33 ± 0.59	1.92 ± 1.48	1.660	0.106	n.s.
TC (mmol/L)	4.63 ± 0.84	4.71 ± 1.05	0.266	0.792	n.s.
HDL-C (mmol/L)	1.33 ± 0.28	6.31 ± 2.25	9.823	0.001	***
LDL-C (mmol/L)	2.78 ± 0.58	2.96 ± 0.84	0.789	0.435	n.s.
HOMA-IR	1.91 ± 1.27	1.72 ± 1.09	0.508	0.615	n.s.
HOMA-IS	0.68 ± 0.30	0.82 ± 0.49	1.090	0.283	n.s.
HOMA-β (%)	93.40 ± 78.84	61.72 ± 42.87	1.579	0.123	n.s.

*SBP* systolic blood pressure, *DBP* diastolic blood pressure, *FBG* fasting blood glucose, *2-h PG* 2-h blood post-load blood glucose, *HbA1c* glycosylated hemoglobin, *FINS* fasting insulin, *UA* uric acid, *TG* triglyceride, *TC* total cholesterol, *HDL-C* high-density lipoprotein cholesterol, *LDL-C* low-density lipoprotein cholesterol, *HOMA-IR* homeostatic model assessment for insulin resistance, *HOMA-IS* homeostatic model assessment for insulin sensibility, *HOMA-β* homeostatic model assessment of β-cell function. ns: not significant, *p < 0.05, **p < 0.01, ***p < 0.001. The Mann–Whitney U test was used to test for significant differences between the groups, data are n (%) or mean ± SD

The correct Table 3 is:

**Table 3:** Comparison of the glycolipid-related indicators between the normal blood glucose and PreDM group among the PXSK patients

	NDMPXSK	PDMPXSK	*t/z*	*p*	
SBP (mmHg)	112.10 ± 7.13	122.30 ± 11.50	3.371	0.002	**
DBP (mmHg)	73.85 ± 6.08	77.85 ± 10.07	1.521	0.137	n.s.
FPG (mmol/L)	5.40 ± 0.39	5.82 ± 0.66	1.458	0.153	n.s.
2hPG (mmol/L)	5.57 ± 0.92	8.88 ± 1.26	9.489	0.001	***
HbA1c (%)	4.76 ± 0.37	5.46 ± 0.49	5.100	0.001	***
FINS (μU/mL)	8.04 ± 5.58	6.60 ± 3.92	0.944	0.351	n.s.
UA (mmol/L)	363.90 ± 88.36	295.48 ± 104.47	2.236	0.031	*
TG (mmol/L)	1.33 ± 0.59	1.92 ± 1.48	1.660	0.106	n.s.
TC (mmol/L)	4.63 ± 0.84	4.71 ± 1.05	0.266	0.792	n.s.
HDL-C (mmol/L)	1.33 ± 0.28	1.65 ± 0.37	2.498	0.017	*
LDL-C (mmol/L)	2.78 ± 0.58	2.96 ± 0.84	0.789	0.435	n.s.
HOMA-IR	1.91 ± 1.27	1.72 ± 1.09	0.508	0.615	n.s.
HOMA-IS	0.68 ± 0.30	0.82 ± 0.49	1.090	0.283	n.s.
HOMA-β (%)	93.40 ± 78.84	61.72 ± 42.87	1.579	0.123	n.s.

*SBP* systolic blood pressure, *DBP* diastolic blood pressure, *FBG* fasting blood glucose, *2-h PG* 2-h blood post-load blood glucose, *HbA1c* glycosylated hemoglobin, *FINS* fasting insulin, *UA* uric acid, *TG* triglyceride, *TC* total cholesterol, *HDL-C* high-density lipoprotein cholesterol, *LDL-C* low-density lipoprotein cholesterol, *HOMA-IR* homeostatic model assessment for insulin resistance, *HOMA-IS* homeostatic model assessment for insulin sensibility, *HOMA-β* homeostatic model assessment of β-cell function. ns: not significant, *p < 0.05, **p < 0.01, ***p < 0.001. The Mann–Whitney U test was used to test for significant differences between the groups, data are n (%) or mean ± SD

The incorrect Table 4 is:

**Table 4:** Comparison of the glycolipid-related indicators between the normal blood glucose group and PreDM group among the SRYP patients

	NDMSRYP n = 20 (Male: 9 Female: 11)	PDMSRYP n = 20 (Male: 12 Female: 8)	t/z	*p*	
SBP (mmHg)	114.00 ± 13.24	120.20 ± 10.89	1.617	0.114	n.s.
DBP (mmHg)	75.90 ± 10.18	78.40 ± 7.76	0.873	0.388	n.s.
FBG (mmol/L)	5.35 ± 0.33	6.04 ± 0.55	4.811	0.001	***
2 h PG (mmol/L)	5.69 ± 1.13	9.12 ± 1.21	9.265	0.001	***
HbA1c (%)	4.83 ± 0.29	5.28 ± 0.63	2.902	0.006	**
FINS (μU/mL)	6.38 ± 4.49	9.87 ± 6.13	2.054	0.047	*
UA (mmol/L)	333.90 ± 100.23	353.57 ± 118.89	0.566	0.575	n.s.
TG (mmol/L)	1.99 ± 1.80	1.96 ± 0.87	0.070	0.947	n.s.
TC (mmol/L)	4.32 ± 0.71	4.96 ± 1.24	2.003	0.052	n.s.
HDL-C (mmol/L)	1.25 ± 0.39	1.28 ± 0.37	0.250	0.804	n.s.
LDL-C (mmol/L)	2.86 ± 0.69	3.23 ± 0.97	1.390	0.173	n.s.
HOMA-IR	1.55 ± 1.14	1.69 ± 1.77	0.297	0.768	n.s.
HOMA-IS	0.96 ± 0.56	0.57 ± 0.40	2.534	0.016	*
HOMA-β (%)	66.86 ± 40.66	79.25 ± 50.68	0.853	0.399	n.s.

*SBP* systolic blood pressure, *DBP* diastolic blood pressure, *FBG* fasting blood glucose, *2-h PG* 2-h blood post-load blood glucose, *HbA1c* glycosylated hemoglobin, *FINS* fasting insulin, *UA* uric acid, *TG* triglyceride, *TC* total cholesterol, *HDL-C* high-density lipoprotein cholesterol, *LDL-C* low-density lipoprotein cholesterol, *HOMA-IR* homeostatic model assessment for insulin resistance, *HOMA-IS* homeostatic model assessment for insulin sensibility, *HOMA-β* homeostatic model assessment of β-cell function. ns: not significant, *p < 0.05, **p < 0.01, ***p < 0.001. The Mann–Whitney U test was used to test for significant differences between the groups, data are n (%) or mean ± SD

The correct Table 4 is:

**Table 4:** Comparison of the glycolipid-related indicators between the normal blood glucose group and PreDM group among the SRYP patients

	NDMSRYP	PDMSRYP	*t/z*	*p*	
SBP (mmHg)	114.00 ± 13.24	120.20 ± 10.89	1.617	0.114	n.s.
DBP (mmHg)	75.90 ± 10.18	78.40 ± 7.76	0.873	0.388	n.s.
FPG (mmol/L)	5.35 ± 0.33	6.04 ± 0.55	4.811	0.001	***
2hPG (mmol/L)	5.69 ± 1.13	9.12 ± 1.21	9.265	0.001	***
HbA1c (%)	4.83 ± 0.29	5.28 ± 0.63	2.902	0.006	**
FINS (μU/mL)	6.38 ± 4.49	10.87 ± 6.27	2.606	0.013	*
UA (mmol/L)	333.90 ± 100.23	353.57 ± 118.89	0.566	0.575	n.s.
TG (mmol/L)	1.99 ± 1.80	1.96 ± 0.87	0.070	0.947	n.s.
TC (mmol/L)	4.32 ± 0.71	4.96 ± 1.24	2.003	0.052	n.s.
HDL-C (mmol/L)	1.25 ± 0.39	1.28 ± 0.37	0.250	0.804	n.s.
LDL-C (mmol/L)	2.86 ± 0.69	3.23 ± 0.97	1.390	0.173	n.s.
HOMA-IR	1.55 ± 1.14	2.95 ± 1.79	0.950	0.005	**
HOMA-IS	0.96 ± 0.56	0.53 ± 0.40	2.794	0.008	**
HOMA-β (%)	66.86 ± 40.66	86.88 ± 53.56	1.331	0.191	n.s.

*SBP* systolic blood pressure, *DBP* diastolic blood pressure, *FBG* fasting blood glucose, *2-h PG* 2-h blood post-load blood glucose, *HbA1c* glycosylated hemoglobin, *FINS* fasting insulin, *UA* uric acid, *TG* triglyceride, *TC* total cholesterol, *HDL-C* high-density lipoprotein cholesterol, *LDL-C* low-density lipoprotein cholesterol, *HOMA-IR* homeostatic model assessment for insulin resistance, *HOMA-IS* homeostatic model assessment for insulin sensibility, *HOMA-β* homeostatic model assessment of β-cell function. ns: not significant, *p < 0.05, **p < 0.01, ***p < 0.001. The Mann–Whitney U test was used to test for significant differences between the groups, data are n (%) or mean ± SD

The original article [1] has been corrected.

## References

[1] Lan Q, Li X, Fang J, et al. Comprehensive biomarker analysis of metabolomics in different syndromes in traditional Chinese medical for prediabetes mellitus. Chin Med. 2024;19:114. 10.1186/s13020-024-00983-1. 39183283 10.1186/s13020-024-00983-1 PMC11346218

