# Supplementary material for: Comprehensive biomarker analysis of metabolomics in different syndromes in traditional Chinese medical for prediabetes mellitus
Source: Chin Med. 2024 Aug 25;19:114. doi: 10.1186/s13020-024-00983-1 (PMC11346218; doi:10.1186/s13020-024-00983-1)
Supplement: Supplementary file 1 — Supplementary Material 1 [file 13020_2024_983_MOESM1_ESM.pdf]

Supplementary information

Comprehensive biomarker analysis of metabolomics in different syndromes in traditional Chinese medical for prediabetes mellitus

Qin Lan[1,5], Xue Li[2], Jianhe Fang[3], Xinyu Yu[4], Zhanxuan E. Wu[2], Caiyun Yang[6], Hui Jian[1], Fei Li[2]

[1] Jiangxi University of Traditional Chinese Medicine, Nanchang 330004, China;

[2] Department of Gastroenterology & Hepatology, Laboratory of Metabolomics and Drug-induced Liver Injury, Frontiers Science Center for Disease-Related Molecular Network, and State Key Laboratory of Respiratory Health and Multimorbidity, West China Hospital, Sichuan University, Chengdu, Sichuan 610041, China;

[3] Medical Ancient Literature Teaching and Research Office, Jiangxi University of Traditional Chinese Medicine, Nanchang 330004, China;

[4] Discipline of Chinese and Western Integrative Medicine, Jiangxi University of Chinese Medicine, Nanchang, 330004, China;

[5] Outpatient Department, Hongdu Traditional Chinese Medicine Hospital Affiliated to Jiangxi University of Traditional Chinese Medicine, Nanchang, 330006, China;

[6] Endocrinology Department II, Hongdu Traditional Chinese Medicine Hospital Affiliated to Jiangxi University of Traditional Chinese Medicine, Nanchang, 330006, China;

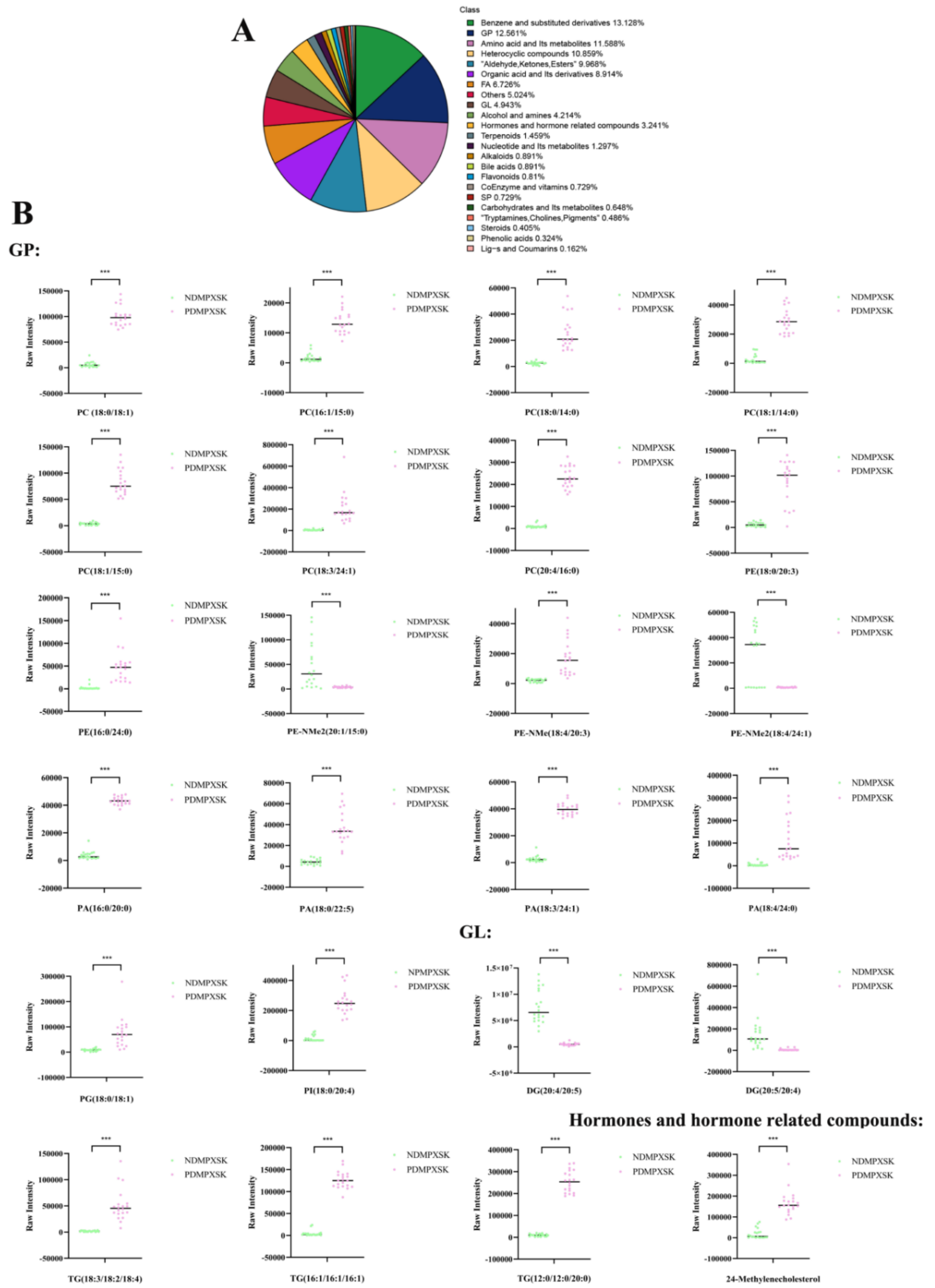

Fig S1 Metabolic of patients with syndrome of PDMPXSK vs NDMPXSK, related to Figures 1.

(A) Class classification chart for differential metabolites. (B) Column chart of potential plasma markers in patients with PDMPXSK vs NDMPXSK. The central line was the median, points were individual observations, Y-axis represents the intensity of metabolite.

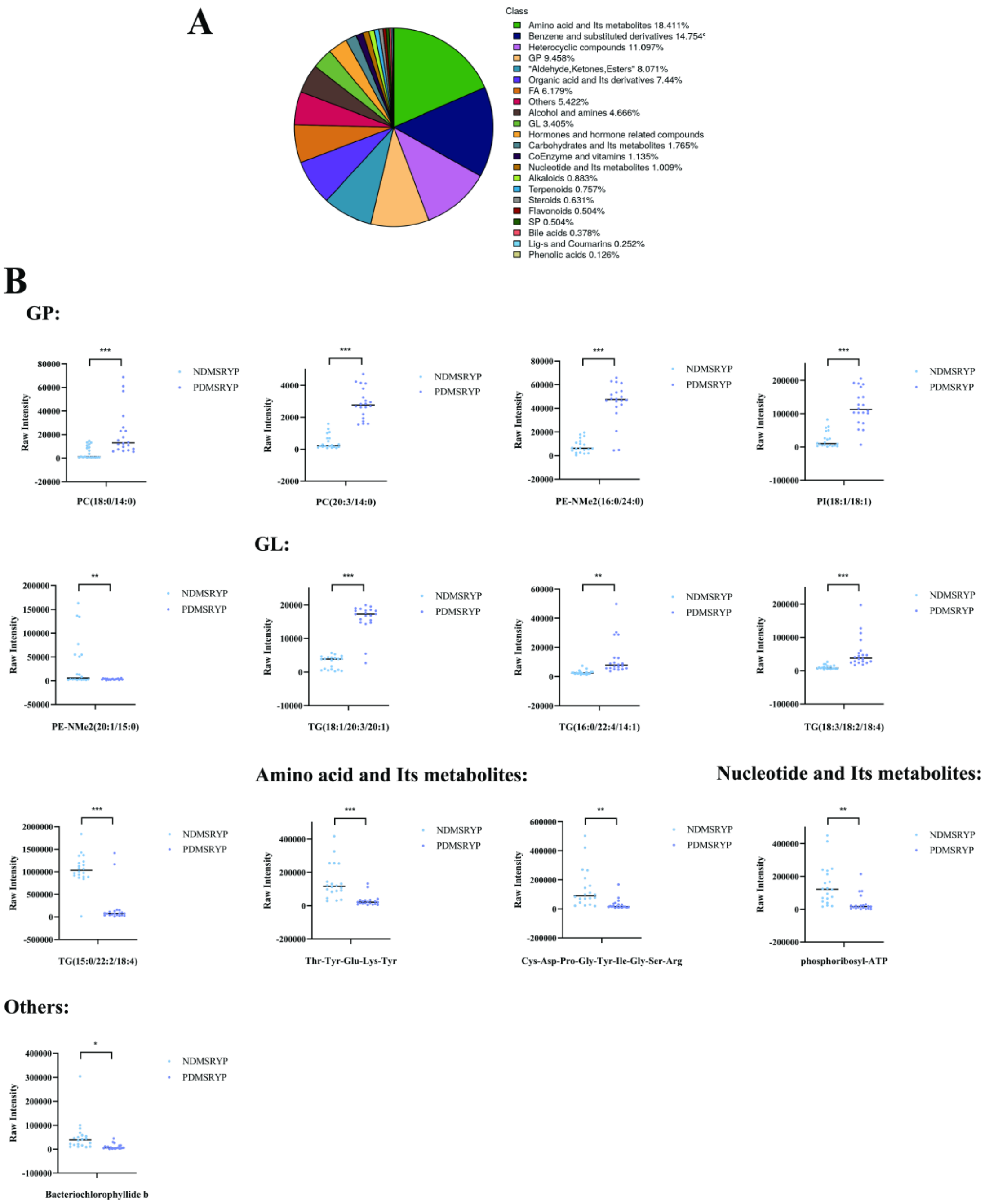

Fig S2 Metabolic of patients with syndrome of PDMSRYP vs NDMSRYP, related to Figures 2.

(A) Class classification chart for differential metabolites. (B) Column chart of potential plasma markers in patients with the PDMSRYP vs NDMSRYP. The centre line was the median, points were individual observations, Y axis was raw intensity of metabolite.

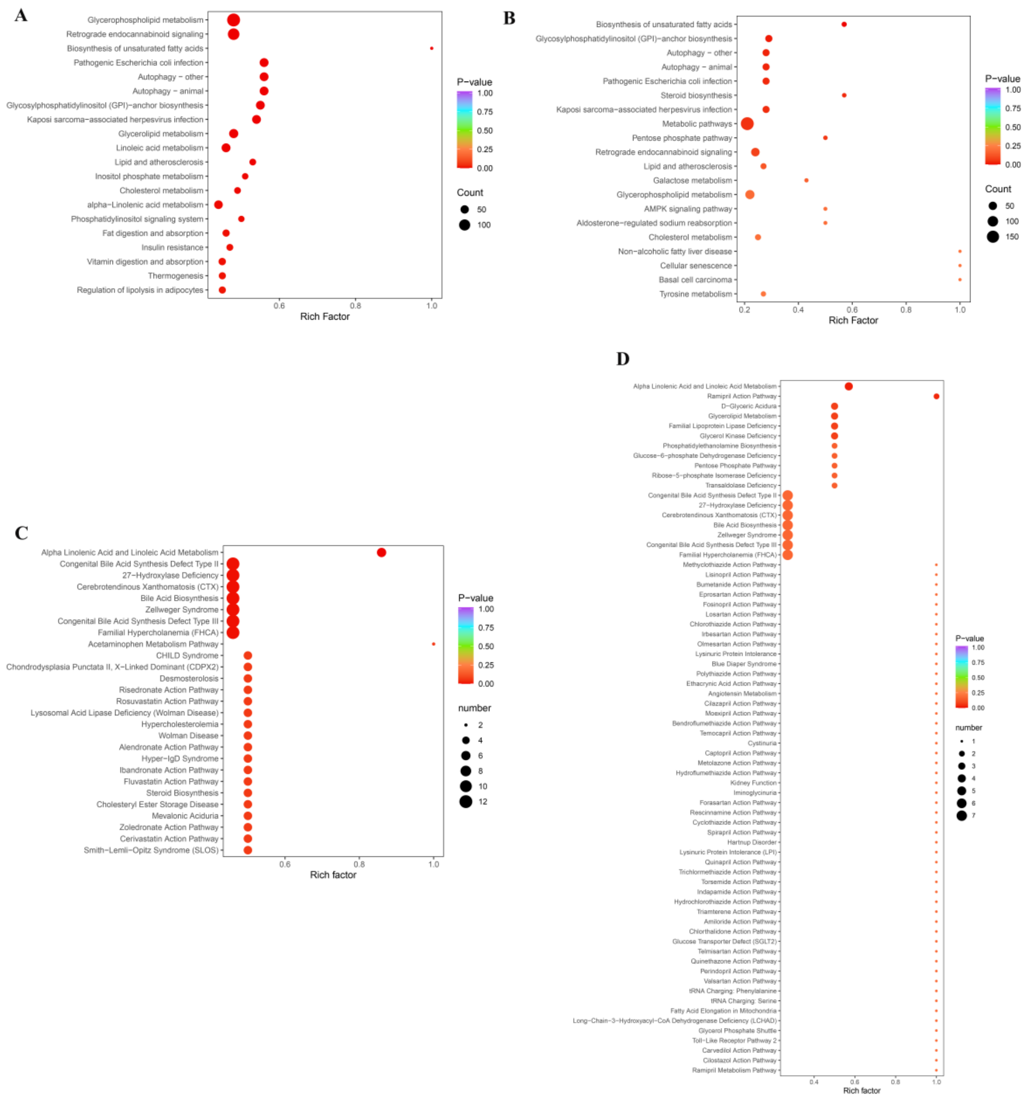

Fig. S3 Plasma metabolite pathway enrichment in the PDMPXSK group and the PDMSRYP group compared to their respective normglyceamic controls, related to Figures 3.

(A) Top 20 KEGG pathways in patients with the PDMPXSK vs NDMPXSK. (B) Top 20 KEGG pathways in patients with the PDMSRYP vs NDMSRYP. (C) SMPDB pathways in patients with the PDMPXSK vs NDMPXSK. (D) SMPDB pathways in patients with the PDMSRYP vs NDMSRYP. Green squares indicate that this metabolite was decreased in pathway, red squares indicate that this metabolite was increased in pathway.

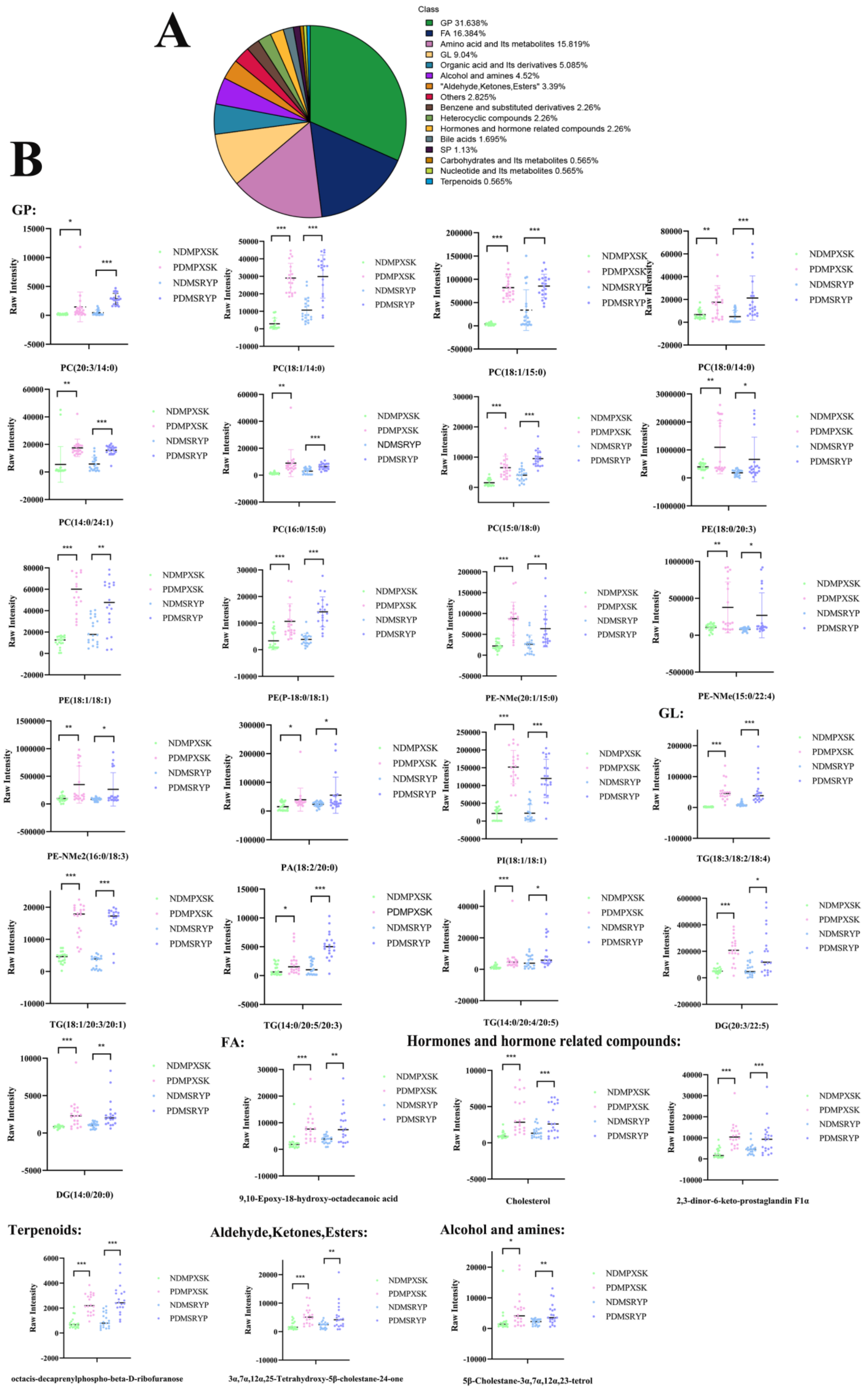

A
Class
GP 31.638%
FA 16.384%
Amino acid and Its metabolites 15.819%
GL 9.04%
Organic acid and Its derivatives 5.085%
Alcohol and amines 4.52%
"Aldehyde,Ketones,Esters" 3.39%
Others 2.825%
Benzene and substituted derivatives 2.26%
Heterocyclic compounds 2.26%
Hormones and hormone related compounds 2.26%
Bile acids 1.695%
SP 1.13%
Carbohydrates and Its metabolites 0.565%
Nucleotide and Its metabolites 0.565%
Terpenoids 0.565%
B
GP:
Raw Intensity
NDMPXSK
PDMPXSK
NDMSRYP
PDMSRYP
PC(20:3/14:0)
PC(18:1/14:0)
PC(18:1/15:0)
PC(18:0/14:0)
PC(14:0/24:1)
PC(16:0/15:0)
PC(15:0/18:0)
PE(18:0/20:3)
PE(18:1/18:1)
PE(P-18:0/18:1)
PE-NMe(20:1/15:0)
PE-NMe(15:0/22:4)
PE-NMe2(16:0/18:3)
PA(18:2/20:0)
PI(18:1/18:1)
GL:
TG(18:3/18:2/18:4)
TG(18:1/20:3/20:1)
TG(14:0/20:5/20:3)
TG(14:0/20:4/20:5)
DG(20:3/22:5)
DG(14:0/20:0)
FA:
9,10-Epoxy-18-hydroxy-octadecanoic acid
Hormones and hormone related compounds:
Cholesterol
2,3-dinor-6-keto-prostaglandin F1α
Terpenoids:
octacis-decaprenylphospho-beta-D-ribofuranose
Aldehyde,Ketones,Esters:
3α,7α,12α,25-Tetrahydroxy-5β-cholestane-24-one
Alcohol and amines:
5β-Cholestane-3α,7α,12α,23-tetrol

Fig S4 The common difference metabolites of the PDMPXSK and the PDMSRYP, related to Figures 4.

(A) Class classification chart for differential metabolites. (B) Column chart of potential elevated plasma markers in PreDM patients. The centre line was the median, points were individual observations, Y axis was raw intensity of metabolite.

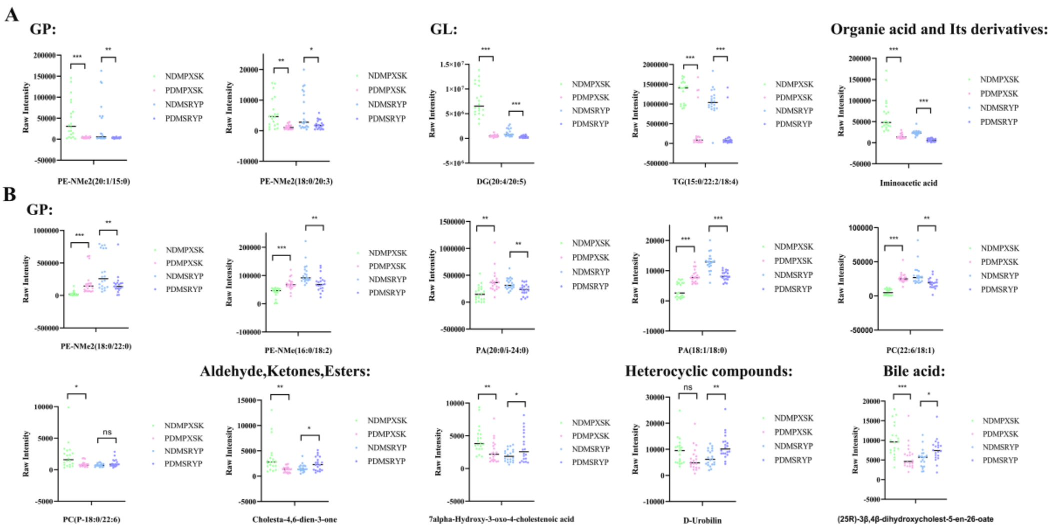

Fig S5 The common metabolite makers of PreDM with PXSK syndrome and PreDM with SRYP syndrome, related to Figures 4.

(A) Column chart of potential reduced plasma markers in PreDM patients. The centre line was the median, points were individual observations, Y axis was raw intensity of metabolite. (B) Column chart of potential plasma markers with opposite changes in the PDMPXSK vs NDMPXSK and the PDMSRYP vs NDMSRYP. The centre line was the median, points were individual observations, Y axis was raw intensity of metabolite.

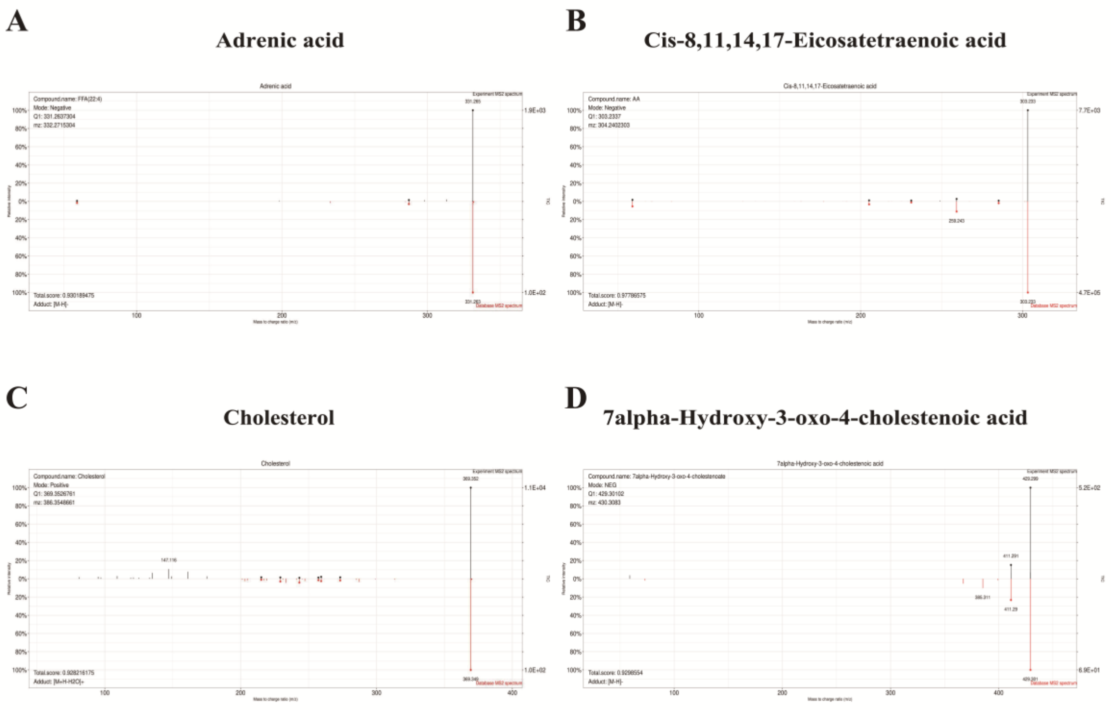

Fig S6 The MS/MS spectra of typical identified metabolite.

(A) Adrenic acid, (B) Cis-8,11,14,17-Eicosatetraenoic acid, (C) Cholesterol, (D) 7alpha-Hydroxy-3-oxo-4-cholestenoic acid.
